# A physiological perspective on the origin and evolution of photosynthesis

**DOI:** 10.1093/femsre/fux056

**Published:** 2017-11-21

**Authors:** William F Martin, Donald A Bryant, J Thomas Beatty

**Affiliations:** 1 Institute for Molecular Evolution, University of Düsseldorf, D-40225 Düsseldorf, Germany; 2 Department of Biochemistry and Molecular Biology, The Pennsylvania State University, University Park, PA 16802, USA; 3 Department of Chemistry and Biochemistry, Montana State University, Bozeman, MT 59717, USA; 4 Department of Microbiology and Immunology, University of British Columbia, 2350 Health Sciences Mall, Vancouver, BC, V6T 1Z3, Canada

**Keywords:** hydrothermal light, Zn-tetrapyrroles, photothiotrophy, cyanobacteria, lateral gene transfer, reaction center evolution

## Abstract

The origin and early evolution of photosynthesis are reviewed from an ecophysiological perspective. Earth's first ecosystems were chemotrophic, fueled by geological H_2_ at hydrothermal vents and, required flavin-based electron bifurcation to reduce ferredoxin for CO_2_ fixation. Chlorophyll-based phototrophy (chlorophototrophy) allowed autotrophs to generate reduced ferredoxin without electron bifurcation, providing them access to reductants other than H_2_. Because high-intensity, short-wavelength electromagnetic radiation at Earth's surface would have been damaging for the first chlorophyll (Chl)-containing cells, photosynthesis probably arose at hydrothermal vents under low-intensity, long-wavelength geothermal light. The first photochemically active pigments were possibly Zn-tetrapyrroles. We suggest that (i) after the evolution of red-absorbing Chl-like pigments, the first light-driven electron transport chains reduced ferredoxin via a type-1 reaction center (RC) progenitor with electrons from H_2_S; (ii) photothioautotrophy, first with one RC and then with two, was the bridge between H_2_-dependent chemolithoautotrophy and water-splitting photosynthesis; (iii) photothiotrophy sustained primary production in the photic zone of Archean oceans; (iv) photosynthesis arose in an anoxygenic cyanobacterial progenitor; (v) Chl *a* is the ancestral Chl; and (vi), anoxygenic chlorophototrophic lineages characterized so far acquired, by horizontal gene transfer, RCs and Chl biosynthesis with or without autotrophy, from the architects of chlorophototrophy—the cyanobacterial lineage.

## Teaser

Questions of how and where chlorophyll-based photosynthesis (chlorophototrophy) arose and how the process subsequently spread among bacteria are typically investigated using phylogenetic trees, but in prokaryotes horizontal gene transfer decouples physiology from phylogeny; here we address the evolution of photosynthesis not from the perspective of gene or lineage phylogenies, but from the physiological perspective of chemical processes.

## INTRODUCTION

Autotrophs have fueled primary production on Earth for at least 3.95 billion years (Tashiro *et al.*^2017^). The advent of photosynthesis—light-dependent CO_2_ fixation—was a pivotal event in microbial evolution, yet several key aspects of its origin remain unresolved. Photosynthesis encompasses two discrete physiological processes: chlorophototrophy, the use of chlorophyll (Chl) and light to generate ATP and/or reducing power, and the fixation of CO_2_ for biomass production and growth. Photosynthesis furthermore exists in two basic forms: anoxygenic photosynthesis involving one reaction center (RC) and oxygen-producing photosynthesis involving two. There is broad consensus that H_2_-based chemosynthesis predated chlorophototrophy in microbial evolution and that anoxygenic photosynthesis predated the oxygenic form. There is, however, no consensus concerning the physiological processes that mediated either transition. This review will focus on the two main transitions in photosynthesis evolution: (i) the origin of chlorophototropy, including the advent of Chl itself as well as key physiological constraints that may have helped the first Chl-bearing cells to access reductants other than H_2_ for primary production, and (ii) physiological intermediate states in the emergence of oxygenic photosynthesis from simpler anoxygenic versions.

Gene phylogenies offer limited insight into the matter because phylogenies are inherently error-prone (Williams *et al.*^2013^; Graur 2016) and because horizontal gene transfer has played a substantial role in generating the highly dispersed distribution of photosynthesis that is observed among modern bacterial lineages (Fischer, Hemp and Johnson 2016). Moreover, there is no consensus concerning either the numbers of transfers that took place during evolution or the directions in which those transfers occurred (Olson and Pierson 1987; Baymann *et al.*^2001^).

The evolution of photosynthesis has been amply reviewed in recent years (*inter alia*, Xiong and Bauer 2002a, 2002b; Allen 2005; Björn and Govindjee 2009; Hohmann-Mariott and Blankenship 2011; Williamson *et al.*^2011^; Fischer, Hemp and Johnson 2016), so there is no need for a new overview. Yet there is room to consider specific aspects of the physiological and ecological setting for the origin of photosynthesis, the subsequent evolution of reaction centers (RC), their co-evolution (or not) with chlorophyll (Chl) biosynthesis and CO_2_ fixation pathways, and the nature and role of low-potential electron donors. Phylogeny in the sense of prokaryotic lineage relationships (Fox *et al.*^1980^) is not our focus because photosynthesis arose and evolved in bacteria, and horizontal gene transfer in prokaryotes often decouples physiology from phylogeny (Wagner *et al*. 2017a).

## THE PROBLEM

The basic puzzle concerning the evolution of photosynthesis on the basis of currently known Chl-harboring prokaryotic lineages is summarized in Table 1. The two types of RC, type-1 RC or RC1 and type-2 RC or RC2 (Golbeck 1993; Schubert *et al.*^1998^; Sadekar, Raymond and Blankenship 2006), may be subdivided further into four types of RC. There are homodimeric types of RC1 (in the green sulfur bacteria *Chlorobia*, *Heliobacteriaceae* and *Acidobacteria*) and heterodimeric types of RC1 (photosystem I, or PSI, in *Cyanobacteria*), and there are two types of RC2 (in *Proteobacteria, Chloroflexi* and *Gemmatimonadetes*; and photosystem II (PSII) of *Cyanobacteria*) that are both heterodimeric (Hohmann-Marriott and Blankenship 2011; Fischer, Hemp and Johnson 2016). The RCs are combined with three of the six known pathways of CO_2_ fixation (Fuchs 2011), and there are seven bacterial phyla within which Chl-based phototrophy occurs (Fischer, Hemp and Johnson 2016). Three of those lineages (*Acidobacteria, Chlorobi* and *Chloroflexi*) produce chlorosomes (Bryant and Liu 2013), antenna complexes containing self-assembling nanotubular, bacteriochlorophyll (BChl) suprastructures with a protein-stabilized, lipid monolayer envelope. Six of the phyla have members that grow aerobically (*Acidobacteria, Chlorobi, Chloroflexi, Proteobacteria, Gemmatimonadetes* and *Cyanobacteria*). Three lineages lack CO_2_ fixation pathways altogether (*Firmicutes*/heliobacteria, *Acidobacteria* and *Gemmatimonadetes*). Four lineages harbor photolithoautotrophic forms, whereas the remainder grow photoheterotrophically or photomixotrophically. The photolithoautotrophic Fe^2+^-oxidizing (photoferrotrophic) *Rhodopseudomonas palustris* strain TIE1 (Jiao *et al.*^2005^) is a notable exception among the *Proteobacteria*, which otherwise are notoriously versatile regarding their use of the Calvin-Benson-Bassham (CBB) cycle under aerobic or anaerobic conditions, in the light or in the dark, using H_2_, H_2_S or organic compounds as electron sources (Madigan and Gest 1979; McKinlay and Harwood 2010).

**Table 1.** Some evolutionarily relevant physiological properties of chlorophyll-containing phototrophic bacteria.

RC type	Taxon	PS^a^	Aerobic^b^ heterotrophs	CO_2_ fixation^c^	Chlorosomes^d^	Photolithoautotrophy, e^−^ donor
1	*Firmicutes*		No^e^			
	*Heliobacterium*	An	No	No	–	No
1	*Acidobacteria*		Yes^f^			
	*Chloracidobacterium*	μOx	Yes	No	+	No
1	*Chlorobi*		Yes^g^			
	*Chlorobium*	An	No	rTCA	+	H_2_S, S^0^, S_2_O_3_^2–^
	*C. ferrooxidans*	An	No	rTCA^h^	+	Fe^2+^
2	*Chloroflexi*		Yes^i^			
	*Chloroflexus*	An	Yes	3HPB^c^	+	H_2_ or H_2_S
	*Oscillochloris*	An	?^j^	CBB	+	H_2_ or H_2_S
2	*Proteobacteria*		Yes^k^			
	Purple sulfur^l^	An	Yes^m^	CBB	–	H_2_S, S^0^, S_2_O_3_^2–^
	Purple non-sulfur^n^	An	Yes^o^	CBB	–	H_2_, H_2_S, S_2_O_3_^2–^
	*Rps. palustris* TIE1^p^	An	Yes	CBB	–	Fe^2+^
	Aerobic anoxygenic^q^	Ox	Yes	No	–	No
2	*Gemmatimonadetes*					
	*Gemmatimonas*	Ox	Yes^r^	No	–	No
1 + 2	*Cyanobacteria*	Ox	Yes	CBB^s^	–	H_2_S^t^ or H_2_O
	*Oscillatoria*	An^u^	Yes	CBB	–	H_2_S^v^ or H_2_O
	*Microcoleus*	An	Yes	CBB	–	H_2_S^v^ or H_2_O

a Refers to O_2_ tolerance during phototrophic growth. An: anaerobic; Ox: aerobic; μOx: microoxic.

b ‘Yes’ indicates the ability for aerobic heterotrophic growth in the genus or the strain, or that the ability for aerobic heterotrophic growth is a widespread trait within the group. References in this column refer to the presence of terminal oxidase genes in the genome, or growth in the presence of O_2_.

c CO_2_ fixation pathways that occur in combination with chlorophyll-based phototrophy. rTCA, reverse tricarboxylic acid (or Arnon-Buchanan) cycle; 3HPB, 3-hydroxypropionate bi-cycle; CBB, Calvin-Benson-Bassham cycle. The enzymes catalyzing the reductive steps in the rTCA cycle are ferredoxin-dependent, as in the case of the other two anaerobic pathways of CO_2_ fixation, the acetyl-CoA (or Wood-Ljungdahl) pathway and the dicarboxylate/4-hydroxybutyrate cycle (Fuchs 2011). The enzymes catalyzing the reductive steps in the 3HPB cycle and the CBB cycle are NADPH dependent (Fuchs 2011). We note that of the six pathways of CO_2_ fixation currently known: three have been named by the individuals who characterized them and the other three were characterized by Georg Fuchs.

d The chlorosome protein CsmA, which binds BChl *a* and comprises the baseplate that connects the chlorosome to the FMO protein in GSB and *Cab. thermophilum*, is present in all lineages possessing chlorosomes studied so far (Bryant and Liu 2013) indicating a common ancestry of chlorosome antennae.

e While there are aerobic *Firmicutes*, no phototrophic ones are aerobic. Heliobacteria are strictly anaerobic in phototrophic growth (Heinnickel and Golbeck 2007).

f The acidobacteria were initially describes as aerobes (Kishomoti *et al.*^1991^; Bryant *et al.*^2007^). *Chloracidobacterium thermophilum* was initially described as an aerobe (Bryant *et al.*^2007^; Garcia Costas *et al.*^2012^). It has an absolute O_2_ requirement for growth, but O_2_ levels higher than ∼1% ambient O_2_ inhibit growth (Tank and Bryant 2015).

g As shown in fig. 4.6 in Bryant and Liu (2013), O_2_-reducing terminal oxidases (*bb*_3_, *bd*) occur in most lineages of *Chlorobi*. Moreover, *aa*_3_, *bb*_3_ and *bd* are present in the non-photosynthetic ancestors of the photosynthetic *Chlorobi*. There are aerobic *Chlorobi*, but only ‘*Candidatus* Thermochlorobacter aerophilum’ is a chlorophototroph (Liu *et al.*^2012^; Tank *et al.*^2017^). All members of the family *Chlorobiaceae* are strictly anaerobic photolithoautotrophs.

h The enzymes of the rTCA cycle are present in the draft genome of *C. phaeoferrooxidans* (Crowe *et al.*^2017^) and the bacterium grows autotrophically.

i See Hanada and Pierson (2006). There is another phototroph in one of the deep branching families of the group, ‘*Candidatus* Roseilinea gracile’, that does not belong to the *Chloroflexaceae.* It has type 2 RCs, and BChl *a* but apparently lacks chlorosomes. Its terminal acceptors are not known but it grows as an anaerobe. See Tank *et al.* (2017) and Thiel *et al.* (2017).

j We could not find reports for aerobic heterotrophic growth of *Oscillochloris*, but it branches in phylogenies after *Chloroflexus* and *Roseiflexus*, both of which grow aerobically in the dark (Hanada *et al.*^2002^)

k Most *Proteobacteria* are capable of aerobic heterotrophic growth (Kersters *et al.*^2006^).

l Purple sulfur bacteria can use H_2_S, S^0^ and other sulfur compounds during photoautotrophic growth (Dahl 2017), *Chromatium vinosum* is a well-studied example (Dahl 2017). There are also photoferrotrophic purple sulfur bacteria such as *Thiodictyon* (Hegler *et al.*^2008^; Camacho *et al.*^2017^).

m Several photosynthetic members of the *Chromatiaceae* (purple sulfur) respire oxygen (Overmann and Pfennig 1992). Aerobic growth is present among both *Chromatiaceae* (Imhoff 2006b) and *Ectothiorhodospiraceae* (Imhoff 2006c).

n Some *Rhodobacter* species are able to thrive as photolithoautotrophs, using reduced sulfur compounds (sulfide, thiosulfate) as electron donors (Pujalte *et al.*^2014^). Elemental sulfur (polysulfide) is the common end product of sulfide oxidation, although some species such as *R. veldkampii* can oxidize it to sulfate (Hansen and Imhoff 1985). Several *Rhodopseudomonas* species can use H_2_, H_2_S or S_2_O_3_^2–^ (de Souza *et al.*^2014^).

o The photosynthetic purple non-sulfur bacteria are typically facultative anaerobes (McEwan 1994; Imhoff 2006a).

p *Rhodopseudomonas palustris* strain TIE1 grows photoautotrophically with Fe^2+^ as the electron source (Bird *et al.*^2011^).

q Aerobic anoxygenic phototrophs are found in α, β and γ subclasses of *Proteobacteria* and are very common in modern environments (Yurkov and Beatty 1998; Koblizeck 2015).

r Zeng *et al.* (2014) reported *Gemmatimonas phototrophica* as growing semiaerobically in 10% O_2_ instead of 20% O_2_.

s The Calvin cycle in different bacterial groups entails deeply divergent and in some cases unrelated enzymes, for example, classI/classII aldolase, classI/classII phosphoribulokinase or different forms of RubisCO (Martin and Schnarrenberger 1997).

t See Allen (2005) and Oren and Padan (1978) for light-dependent anoxygenic H_2_S-dependent growth of *O. limnetica* to produce extracellular S^±0^; see De Wit and van Gemerden (1987) and Rabenstein *et al.* (1995) for evidence that cyanobacterial light-dependent H_2_S oxidation can generate thiosulfate (probably via sulfite).

u See the text.

v In most cyanobacteria that perform anoxygenic photosynthesis, sulfide is quantitatively oxidized to thiosulfate, probably via sulfite (de Wit and van Gemerden 1987; Rabenstein *et al.*^1995^).

The chlorophototrophic lineages of prokaryotes are not closely related in any modern phylogenetic scheme (Fischer, Hemp and Johnson 2016), largely because chlorophototrophy has been spread among prokaryotes by horizontal gene transfer during evolution. There are ∼100 kb plasmids in some proteobacteria that contain all genes required for the RC, Chl and carotenoid biosynthesis in a cluster that is collinear with segments of proteobacterial chromosomes, and that is mobile among marine *Roseobacter* strains (Petersen *et al.*^2012^). Functional genes for PSI and PSII are mobile on phage genomes in the marine environment (Fridman, Flores-Uribe and Larom 2017). The most recently characterized chlorophototroph, a member of the phylum *Gemmatimonadetes*, clearly acquired its phototrophic gene cluster from a purple phototrophic bacterium (Zeng *et al.*^2014^). Many of the photosynthetic chlorophototrophic lineages can harness reduced sulfur species as an electron donor for photoautotrophic growth (Table 1), some lineages can use Fe^2+^, but only cyanobacteria can oxidize water. Notably, all of the photosynthetic lineages listed in Table 1 having members that can fix CO_2_ also include members that can use H_2_S as the electron donor during photolithoautotrophic growth. A number of cyanobacteria can grow photosynthetically with H_2_S as the electron source (Oren and Padan 1978; De wit and van Gemerden 1987; Rabenstein, Rethmeier and Fischer 1995; Grim and Dick 2016; Klatt *et al.*^2015^, ^2016^; Miller and Bebout 2004).

## ITS ABOUT REDUCING CO_2_

Of the many things that the emergence of photosynthesis did for life (Judson 2017), perhaps the most important was to increase primary production. Before the origin of photosynthesis, there was only one significant source of electrons to fuel primary production on Earth: geochemical H_2_. Primary production via organic substances from space was not possible because of the paucity of fermentable substrates that have been found in such material and because of their structural heterogeneity, comprising a mixture of different isomers present at parts per billion concentrations each (Schönheit, Buckel and Martin 2016). CO_2_ was thus the starting material for organic biosynthesis, spurring the accumulation and diversification of the first forms of life and first ecosystems.

**Figure 1.**
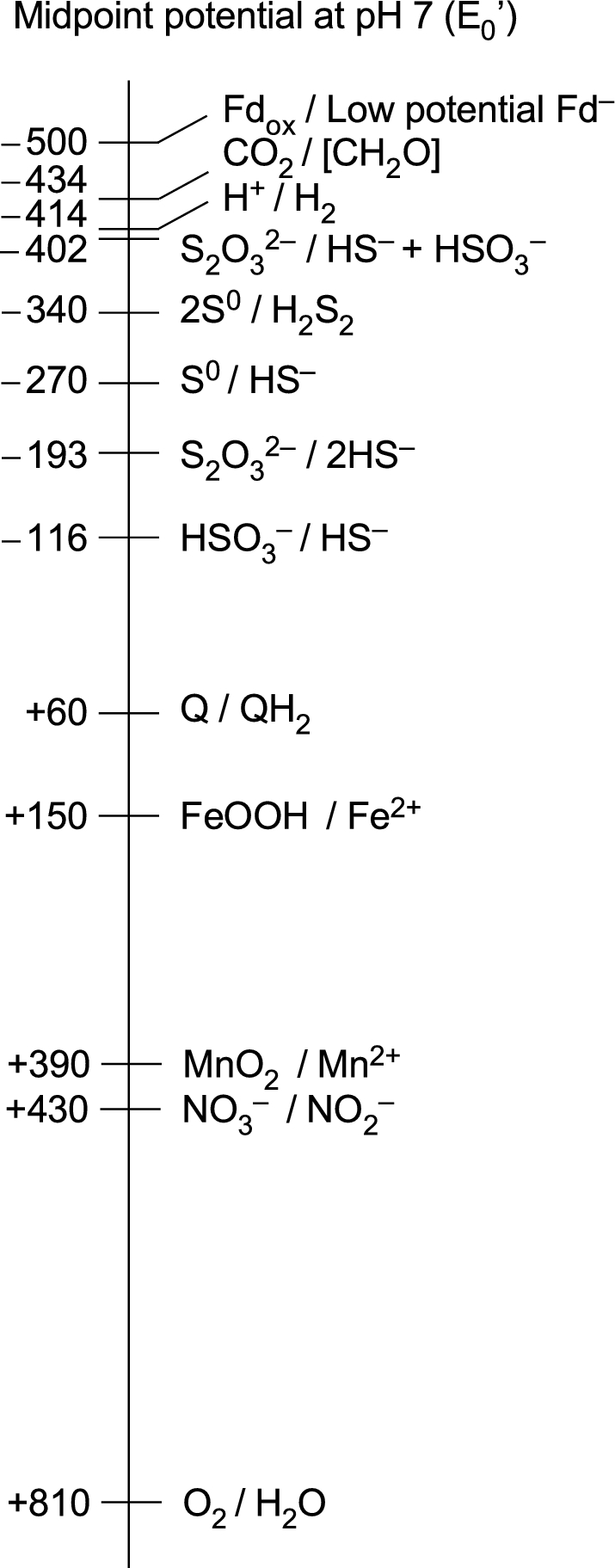
Midpoint potentials of some redox couples relevant to this paper. The relevant redox couples for chemolithoautotrophic primary production are the uppermost three. Values are from Thauer, Jungermann and Decker (1977), Brune (1989), Griffin, Schott and Schink (2007), Sharma *et al.* (2012) and Lengeler, Drews and Schlegel (1999). The H_2_AsO_4_/H_3_AsO_3_ couple (arsenate/arsenite, not shown) has a midpoint potential at pH 7 of +54 mV and is used by some chlorophototrophic proteobacteria (Budinoff and Hollibaugh 2008).

Among the electron donors that were widely available on the early Earth for CO_2_ reduction, only H_2_ has a sufficiently low midpoint potential to support CO_2_ reduction (Fig. 1; Table 2). The exhalation of H_2_ at hydrothermal vents stems from a spontaneous (exergonic) geochemical reaction called serpentinization (Sleep *et al.*^2004^; Martin *et al.*^2008^; Russell, Hall and Martin 2010; Sleep, Bird and Pope 2011). Serpentinization typically generates in the range of 10–20 mM H_2_ in the effluent of modern vents (Kelley, Baross and Delaney 2002; Schrenk, Brazelton and Lang 2013). During serpentinization, water circulating through hydrothermal systems comes into contact with Fe^2+^-bearing minerals in the crust that transfer electrons to water, producing H_2_ and leaving Fe^3+^ minerals such as magnetite (Fe_3_O_4_) behind (Bach *et al.*^2006^; Schrenk, Brazelton and Lang 2013). The serpentinization reaction can be written in a simplified form (Sleep, Bird and Pope 2011; McCollom and Seewald 2013) as

(1) \begin{eqnarray*} 3{\rm{FeO}}\left( {{\rm{in}}\,{\rm{rocks}}} \right) &+& {{\rm{H}}_2}{\rm{O}}\left( {{\rm{liquid}}} \right)\rightarrow{{\rm{H}}_2}\left( {{\rm{in}}\,{\rm{solution}}} \right)\nonumber\\ &+& {\rm{F}}{{\rm{e}}_3}{{\rm{O}}_4}({\rm{magnetite}}) \end{eqnarray*}

with the relevant redox reaction being the oxidation of Fe^2+^ to Fe^3+^ to generate H_2_, which is released into the hydrothermal effluent (Sleep *et al.*^2004^; Bach *et al.*^2006^; Sleep, Bird and Pope 2011; Schrenk, Brazelton and Lang 2013).

**Table 2.** Key transitions in physiological evolution and primary production.

Relative age	Tetrapyrrole^a^	Physiology^a^	Modern group	Electron sources
Modern	Chlorophyll	Chlorophototrophy with RCI and RCII	Cyanobacteria	H_2_S, H_2_O
Advanced	Chlorophyll	Chlorophototrophy with RCI	Cyanobacteria, chlorobia	H_2_S^b^
Intermediate-2	Porphyrins	Mixotrophy	None	H_2_S
Intermediate-1	Heme	Anaerobic respiration	Sulfur reducers^c^, autotrophic ε-proteo^c^	Organics^d^, H_2_
Primordial	Cobalamin^e^	Chemolithoautotrophy	Acetogens, methanogens^f^	H_2_

a Heme before chlorophyll: Granick (1965); cobalamin before heme before chlorophyll: Decker *et al.* (1970). Chemolithoautotrophs before anaerobic respiration before anoyxgenic photosynthesis: Decker *et al.* (1970).

b Before the advent of photosynthetic H_2_S oxidation, H_2_ was the sole reductant driving primary production. Chlorophotosynthetic use of the same reductant (H_2_) would not substantially increase primary production (see text) and would be restricted to environments where H_2_ from serpentinization was discharged in photic environments. Of course, H_2_ is also generated by anaerobic fermentations of reduced carbon compounds, but that does not permit a net increase in primary production.

c Here, the term sulfur reducers designates organisms that gain energy by S, sulfite or sulfate reduction, a very broad definition (Rabus *et al.*^2015^). Many sulfate reducers grow chemolithoautotrophically using the acetyl-CoA pathway for carbon metabolism and sulfate reduction with H_2_ for energy metabolism, or chemoorganoheterotrophically on acetate or lactate (Rabus *et al.*^2015^). H_2_-dependent epsilonproteobacteria that use the rTCA cycle for autotrophy and anaerobic respiration via sulfur reduction, for example, would be another kind of early intermediate. See the text.

d Of course, use of organics as electron donors does not increase primary production, but it increases metabolic flexibility and permits specialization of carbon and energy metabolism.

e Decker *et al.* (1970); Sousa *et al.* (2013).

f In acetogens and methanogens, the acetyl-CoA pathway is the central pathway of carbon assimilation and energy metabolism, whereas the ion gradient that drives ATP-synthase is generated during the process of CO_2_ reduction with electrons from H_2_ (Thauer *et al.*^2008^; Schuchmann and Müller 2014; Sousa and Martin. 2014).

The H_2_ so generated escapes from the crust into the ocean via the circulating hydrothermal vent effluent. Serpentinization has been going on for 4.2 billion years, since there was liquid water on Earth (Sleep, Bird and Pope 2011). In addition to reducing water to H_2_, it also reduces CO_2_ to simple organic compounds such as methane and acetate (McDermott *et al.*^2015^; McCollom 2016; Miller *et al.*^2017^). The serpentinization reactions involving CO_2_ reduction reveal strong similarities between a spontaneous geochemical process and the core physiology of primitive anaerobes that reduce CO_2_ with electrons from H_2_ as their main bioenergetic reaction (Sousa and Martin 2014). Geochemical evidence indicates that anoxygenic photosynthesis was probably in existence by 3.3 to 3.4 billion years ago (Tice and Lowe 2006; Westall *et al.*^2006^, ^2011^; Arndt and Nisbet 2012). It has been estimated that the transition from H_2_-based chemosynthesis to anoxygenic photosynthesis increased Earth's yearly primary production by a factor of ∼2000, and that the subsequent transition to oxygenic photosynthesis increased primary production by an additional factor of ∼30 (Raven 2009). Thus, prior to the origin of photosynthesis, primary production on Earth was very limited in magnitude and restricted to sites of H_2_ emission.

## REDUCED FERREDOXIN BEFORE PHOTOSYNTHESIS

The origin of Chl is the starting point of photosynthetic evolution. Based on the order of biosynthetic precursors in modern pathways (Table 2), Chl arose in cells that could synthesize heme, and heme arose in cells that made cobalamin (Decker, Jungermann and Thauer 1970; Sousa *et al.*^2013^). Note that the primitive lineages of acetogens and methanogens synthesize cobalamin, but lack heme (cytochromes) and quinones (Thauer *et al.*^2008^; Schuchman and Müller 2014). We briefly consider the physiology of the cells within which photosynthesis might have arisen, keeping in mind that before the origin of photosynthesis, primary production was anaerobic and dependent on H_2_ generated by serpentinization in hydrothermal systems.

Primary production in strict anaerobes requires reduced ferredoxin, Fd_red_. The oxygen-sensitive (anaerobic) pathways of CO_2_ fixation—the acetyl-CoA pathway, the reductive (or reverse) TCA (rTCA) cycle and the dicarboxylate/4-hydroxybutyrate cycle—entail one or more Fd-dependent reduction steps and harbor one or more oxygen-sensitive enzymes (Fuchs 2011). The first cells were likely H_2_-dependent chemolithoautotrophs that used the linear, exergonic acetyl-CoA pathway for CO_2_ fixation (Fuchs 2011; Poehlein *et al.*^2012^; Takami *et al.*^2012^; Weiss *et al.*^2016^). The acetyl-CoA pathway, also called the Wood-Ljungdahl pathway, is unique among CO_2_ fixation pathways in that it is exergonic, allowing microbes with primitive redox physiology like acetogens and methanogens to generate protonmotive force for ATP synthesis from CO_2_ reduction (Thauer *et al.*^2008^; Poehlein *et al.*^2012^; Schuchmann and Müller 2014). All other CO_2_ fixation pathways require energetic input in the form of ATP (Fuchs 2011). The core CO_2_-fixing and bioenergetic reaction in acetogens and methanogens (that is, the exergonic synthesis of acetate or methane from H_2_ and CO_2_) is strikingly similar to spontaneous geochemical CO_2_ reduction during serpentinization (Sousa and Martin 2014; McDermott *et al.*^2015^; McCollom 2016; Miller *et al.*^2017^).

There is, however, a crucial mechanistic and energetic hurdle to CO_2_ fixation in acetogens and methanogens. Their CO_2_-reducing enzymes require Fd_red_ with a very low midpoint potential, on the order of −500 mV (Fuchs 2011; Buckel and Thauer 2013). This must be generated using electrons from H_2_, with a midpoint potential of –414 mV. During the reduction of the FeS clusters in low-potential Fd by H_2_, electrons must flow energetically uphill (i.e*.* endergonically). To perform this energetic trick, cells employ a recently discovered mechanism called flavin-based electron bifurcation (Herrmann *et al.*^2008^; Li *et al.*^2008^; Buckel and Thauer 2013; Lubner *et al.*^2017^; Wagner *et al*. 2017), in which the electron pair in H_2_ is first transferred to FAD, a transducer of a two-electron to a one-electron transfer. One electron exits FADH energetically uphill to produce Fd_red_ while the other goes to a sufficiently positive electron acceptor, such as NAD^+^ (in acetogens, *E*^o'^ = –320 mV) or the heterodisulfide CoM–S–S–CoB (in methanogens, *E*^o'^ = –140 mV), so that the overall energetics of the reaction are favorable, allowing it to proceed (Buckel and Thauer 2013).

How did the earliest organisms generate low-potential Fd_red_ for CO_2_ reduction prior to evolutionary origin of electron bifurcation? Comparative genomic data indicate that the first cells had a physiology very similar to that of acetogens and methanogens and arose in a geochemical setting rich in FeS minerals (Weiss *et al.*^2016^), in which the Earth's spontaneous redox chemistry provided FeS minerals with the electron-donating and CO_2_-reducing function of Fd_red_, via serpentinization. In addition, zero valent transition metals such as Fe^0^ readily reduce CO_2_ to methanol (Guan *et al.*^2003^) and acetate (He *et al.*^2010^), they occur naturally in hydrothermal vents, for example as awaruite (Ni_3_Fe) which is a common constituent of serpentinizing systems (McCollom 2016), and they are electron sources for methanogenic growth (Daniels *et al.*^1987^). Early chemolithoautotrophs depended on interactions between rocks, water, metals, and H_2_.

H_2_-dependent primary production based on serpentinzation was stable on geological timescales, and it fueled Earth's first ecosystems. Some sulfate reducers also grow autotrophically using the acetyl-CoA pathway (Rabus *et al.*^2015^), and represent a slightly advanced state relative to acetogens and methanogens in which carbon and energy metabolism are both fueled by the reduction of CO_2_ by H_2_ (Buckel and Thauer 2013). In sulfate reducers that use the acetyl-CoA pathway autotrophically, CO_2_ is fixed while energy is obtained by the reduction of sulfur compounds with electrons from H_2_, or organic donors such as acetate or lactate (Rabus *et al.*^2015^).

With accumulating biomass on primordial Earth, the first heterotrophic metabolisms became possible (Schönheit, Buckel and Martin 2016). Bacterial cells consist of roughly 50%–60% protein, 20% RNA, 10% lipids and 5%–10% saccharides (Neidhardt, Ingraham and Schaechter 1990), and so fermentative breakdown of amino acids, purines and sugars became energetically favorable at low H_2_ partial pressures. These fermentations, along with organoheterotrophic and cytochrome-independent respiration using S^±0^ as the terminal acceptor, as found in heterotrophic *Thermococcales* (Schut, Bridger and Adams 2007; Schut *et al.*^2013^), likely were among the first heterotrophic metabolisms (Schönheit, Buckel and Martin 2016). Photosynthesis arose in ecosystems where primary production was chemolithoautotrophic and where heterotrophs lived by consuming the cell mass of the H_2_-dependent primary producers.

### Coupling Fd reduction to CO_2_ reduction

Thoughts on the origin and early evolution of photoautotrophy as well as the occurrence of ancient light carbon isotopes in the geochemical record (Nisbet and Sleep 2001) have long been associated with ribulose-1,5-bisphosphate carboxylase/oxygenase, RuBisCO (Tabita 1999), the CO_2_-fixing enzyme of the Calvin cycle (also called the Calvin-Benson-Bassham or CBB cycle) and the quantitatively most significant entry point of CO_2_ into the modern carbon cycle (Raven 2009). More recent findings concerning the other five pathways of CO_2_ fixation indicate, however, that anaerobic CO_2_-fixing pathways, in particular the acetyl-CoA pathway, which is ferredoxin-dependent, and the reverse TCA cycle, which is both ferredoxin and NAD(P)H dependent, predate the Calvin cycle (Berg 2011; Fuchs 2011), which is fully NADPH dependent and has no Fd-dependent reactions. Consistent with that view, divergent and likely ancient forms of RuBisCO, called type IV RuBisCO or RuBisCO-like proteins (RLPs), have been shown to function in heterotrophic metabolism in diverse bacteria and archaea (Hanson and Tabita 2001; Tabita *et al.*^2007^; Sato and Atomi 2011; Tabita *et al.*^2008^; Aono *et al.*^2012^).

Such findings suggest that the ancestral function of RubisCO likely arose in a heterotrophic context, having later been co-opted into the CBB cycle for CO_2_ fixation. For example, in *Bacillus* species, RLP functions in a heterotrophic methionine salvage pathway (Ashida, Danchin and Yokota 2005; Ashida *et al.*^2003^) and the RLPs from methanogenic archaea, which populate the root in phylogenetic trees of RuBisCO sequences, incorporate ribulose bisphosphate-carboxylase activity into methanogen metabolism (Kono *et al.*^2017^), even though CO_2_ fixation in methanogens proceeds through the acetyl-CoA pathway (Thauer *et al.*^2008^; Fuchs 2011). Perhaps more revealing is the role of RuBisCO activity in the utilization of RNA as a substrate. The first heterotrophs likely lived on the cell mass of dead chemoautotrophs, with RNA as an important source of organic material.

Cells are roughly 20% RNA by weight (Neidhardt, Ingraham and Schaechter 1990). For the first heterotrophs, RNA was an excellent carbon and energy source (Schönheit, Buckel and Martin 2016). In the archaeon *Thermococcus kodakarensis*, RuBisCO participates in a short RNA degradation pathway (Aono *et al.*^2012^), the first enzyme of which phosphorolytically cleaves the base from ribonucleoside monophosphates (RNA breakdown products) to generate ribose-1,5-bisphosphate. The next step is catalyzed by an isomerase that generates ribulose-1,5-bisphosphate, which is cleaved by RuBisCO via carboxylation, generating two molecules of 3-phospho-D-glycerate (3PGA) for carbon and energy metabolism (Sato, Atomi and Imanaka 2007; Aono *et al.*^2012^; Schönheit, Buckel and Martin 2016). Although central carbon and energy metabolism in archaea is very different from that in bacteria (Reher *et al.*^2010^; Bräsen *et al.*^2014^), 3PGA is a universal metabolite among free-living cells, and the same RuBisCO like protein is also found in anaerobic heterotrophic bacteria that lack a CBB cycle (Wrighton *et al.*^2012^).

Because all forms of anoxygenic photosynthesis entail electron transport chains containing cytochromes and quinones, the cells that evolved photosynthesis must have been capable of some sort of respiration involving cytochromes and quinones (Decker, Jungermann and Thauer 1970; Xiong and Bauer 2002a, 2002b). This is consistent with the view that Chl biosynthesis arose subsequent to heme biosynthesis by pathway extension starting from late intermediates, in a specific evolutionary lineage (Granick 1965; Decker, Jungermann and Thauer 1970) (Table 2). The type of respiration is immaterial here, but it was before the advent of O_2_, and hence it cannot have involved high-potential acceptors like O_2_ (Fig. 1). SO_2_, a gas commonly emitted by volcanic activity, was present in the Earth's most ancient oceans, dissolved as sulfite SO_3_^2–^ (Halevy, Zuber and Schrag 2007), and could have been an early terminal electron acceptor. The energy-conserving segment of sulfate reduction starts from sulfite (Rabus *et al.*^2015^; Santos *et al.*^2015^); sulfite (sulfate) reduction and sulfur-based respirations, which generate H_2_S, were likely among the first to arise in metabolic evolution (Decker, Jungermann and Thauer 1970; Arndt and Nisbet 2012). Isotopic signatures from rocks 3.8 to 2.7 billion years of age indicate that the sulfur cycle was operating in a more or less modern form before the rise of oxygen (Grassineau *et al.*^2006^).

Sulfate reducers are replete with cytochromes and quinones (Rabus *et al.*^2015^). Many oxidize fermentation end products such as acetate and lactate for energy metabolism while using the acetyl-CoA pathway for carbon metabolism. Although we use the vernacular term ‘sulfate reducers’ here, in no passage does this wording necessitate the presence or involvement of sulfate. Sulfate reducers are thought to first activate sulfate to sulfite at the expense of ATP and a reductant (Santos *et al.*^2015^), whereas the subsequent six-electron reduction of sulfite to sulfide is exergonic if the electrons stem from H_2_ or organic compounds (Rabus *et al.*^2015^). From the physiological and energetic standpoints on ancient Earth, sulfate reduction is better seen as sulfite reduction. The heme- and cytochrome-containing anaerobes that evolved photosynthesis were most likely facultative chemoautotrophs/chemoheterotrophs. Perhaps they were similar in physiology to sulfate reducers that use the acetyl-CoA pathway (Rabus *et al.*^2015^), or to epsilonproteobacteria that reduce S^0^ using polysulfide reductase and fix CO_2_ by the rTCA cycle (Grote *et al.*^2012^).

The transition to phototrophic Fd reduction may have taken place in a cell that used the rTCA cycle. The rTCA cycle, also called the Arnon-Buchanan cycle, consumes ATP and thus must be supported by an independent energy metabolism generating ATP (Fuchs 2011), which is in contrast to a central role in generating ATP as in the acetyl-CoA pathway during chemoautotrophic growth. Separate pathways of carbon and energy metabolism would have been conducive to the onset of phototrophy. The incomplete 'horseshoe' (i.e*.* branched) TCA cycle manifest in acetogens and methanogens (Fuchs 1989; Simpson and Whitman 1993; Furdui and Ragsdale 2000) would be ancestral to the rTCA cycle (Martin and Russell 2007). The TCA cycle can function as a branched pathway in *Escherichia coli* and other chemotrophic bacteria during anaerobic fermentative growth (and largely during aerobic growth on glucose as well; Neidhardt, Ingraham and Schaechter 1990), whereas anaerobic phototrophs such as *Rhodobacter* can use light-driven, reverse electron transfer to catalyze the succinate dehydrogenase reaction and thereby run a complete, oxidative TCA cycle anaerobically (Beatty and Gest 1981). The mechanism of flavin-based electron bifurcation in epsilonproteobacteria that use the rTCA cycle has not been reported, but it could involve electron bifurcation at the heterotrimeric Fe-Fe hydrogenase, HydABC, which catalyzes the reversible reduction of NAD^+^ and Fd with H_2_ in *Thermotoga* spp. (Schut and Adams 2009) and acetogens (Schuchmann and Müller 2014). The evolution of phototrophic Fd reduction in a cell that used the rTCA cycle would have provided a net boost to autotrophic carbon metabolism without directly interfering in energy metabolism. It is worth noting that organisms utilizing the acetyl-CoA pathway and the rTCA cycle are common in hydrothermal vent environments (Chapelle *et al.*^2002^; Campbell and Cary 2004; Takai *et al.*^2005^; Lever *et al.*^2010^; Lever 2012).

## ZN-TETRAPYRROLE TRIPLET AND FERREDOXIN REDUCTION

Primary productivity was limited by H_2_ production at vents until the origin of photosynthesis. Geochemical data indicate that photosynthesis-based primary production was in operation some 3.4 billion years ago (Tice and Lowe 2006; Westall *et al.*^2006^, ^2011^), long before the appearance of O_2_ roughly 2.4 billion years ago (Arndt and Nisbet 2012; Lyons, Reinhard and Planavsky 2014; Fischer, Hemp and Johnson 2016). It is widely agreed that anoxygenic photosynthesis preceded oxygenic photosynthesis, but how and where anoxygenic photosynthesis arose has been unresolved.

From our standpoint, the main initial benefit of anoxygenic photosynthesis was not the additional energy provided by cyclic electron flow using an RC2 to produce protonmotive force for ATP synthesis. Instead, the main benefit was that anoxygenic photosynthesis provided access to a new source of moderately low-potential electrons—i.e. from a donor other than H_2_—that could be used together with light energy to generate Fd_red_ for the purpose of CO_2_ fixation. Therefore, the RC1, which provides linear electron flow to Fd, came first. Given that H_2_ was the lowest potential sustainable source of electrons on the early Earth, the only way to convert electrons from a higher potential donor to a much lower potential is, as far as we know, by harnessing light to generate electrons of sufficiently low potential to reduce Fd to Fd_red_. To rephrase, for emphasis: in a world where H_2_ was the electron donor with the most negative midpoint potential (Fig. 1), Chl-based phototrophy provided an alternative mechanism to flavin-based electron bifurcation as a means to generate low-potential Fd_red_ for CO_2_ fixation.

As a short digression, and small caveat to the foregoing sentence, one might imagine that flavin-based electron bifurcation could, in principle, offer a mechanism to generate low-potential Fd_red_ with electrons from donors with more positive midpoint potentials than H_2_, such as H_2_S or other reduced sulfur species, provided that very high potential acceptors (such as O_2_) were available in the environment prior to the origin of photosynthesis. However, there is no evidence for the existence on the early Earth of very high potential acceptors with midpoint potentials near or exceeding that of O_2_ (+810 mV) in amounts approaching those required to run an ecosystem. In that context, one might ask whether the levels of H_2_ generated by serpentinization are really sufficient to fuel chemolithoautotrophs. The answer is yes: the 10–20 mMol/kg levels of H_2_ commonly observed at vents of serpentinizing systems are orders of magnitude more than the H_2_ partial pressure of roughly 10 Pa that methanogens lacking cytochromes require for sustained growth (Thauer *et al.*^2008^). Digression aside, the capability to utilize alternative electron sources would have conferred on cells a powerful selective advantage in H_2_-limited environments.

### Zn-protoporphyrin IX: a functional bridge to Chl

There are two prerequisites for harnessing light energy. One is a source of light, with which we will deal in a subsequent section (focusing not on sunlight, however, but on light that is emitted from hydrothermal vents). The other prerequisite is a molecule with the photochemical properties of Chl, which we consider now. At the time of origin of the Chl biosynthetic pathway, the first enzymes involved were surely not finely tuned, and enzymes in related pathways could have promoted the accumulation of intermediates as side reactions. Preexisting enzymes could have furthermore participated in more than one pathway. Although insertion of Zn^+2^ into protoporphyrin IX (PPIX) occurs spontaneously (Taketani *et al.*^2007^; Becker *et al.*^2012^), the insertion of Zn^+2^ is catalyzed by ferrochelatase (Hunter, Sampson and Ferreira 2008; Chau *et al.*^2011^).

For example, in *bchD* mutants of *Rhodobacter sphaeroides* lacking Mg-chelatase, which catalyzes the first committed step of modern Chl/BChl synthesis, ferrochelatase from the heme biosynthetic pathway inserts Zn^2+^ into PPIX, leading to the production of Zn-PPIX monomethyl ester, Zn-divinyl-protochlorophyllide and Zn-BChl *a* (Jaschke *et al.*^2011^). As Williamson *et al.* (2011) have previously pointed out, and as we further develop in the following sections, Zn-tetrapyrroles have interesting properties in the context of Chl evolution, and we suggest that it is possible that Zn-tetrapyrroles predated Mg-derivatives.

What might cells have done with Zn-PPIX? Zinc typically exhibits five-coordinate geometry in porphyrins (Favereau *et al.*^2015^). Zinc in Zn-PPIX would not have been useful for electron transfer reactions as iron in heme (in cytochromes) because Zn has only one valence state, Zn^+2^, and therefore is biologically redox inert (Kręzèl and Maret 2016). However, if Zn-PPIX were inserted into a preexisting cytosolic heme-binding protein, such as a soluble apocytochrome *b*, that protein, although unable to catalyze cytochrome-type, one-electron transfer reactions, would have been able to *absorb and store light energy* by virtue of its Zn-PPIX chromophore. How so? Absorption of light (∼410, 540 and 580 nm) by Zn-PPIX (Jaschke *et al.*^2011^) produces a long-lived triplet excited state with a yield of ∼90% (Vanderkooi and Berger 1989) and—importantly—with a long lifetime of ∼7 to 15 ms (Dixit, Waring and Vanderkooi 1981).

### Using light to reduce ferredoxin

**Figure 2.**
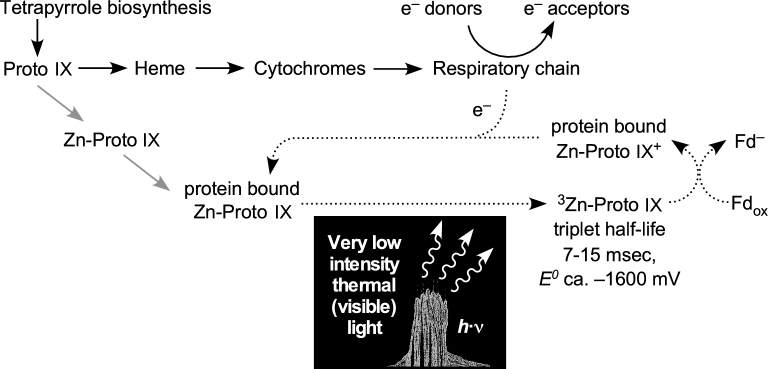
A proposal for the origin of Chl-based phototrophy (see the text). In the primitive, ancestral pathway, a side activity of ferrochelatase produces Zn-PPIX as in modern cells although Zn^2+^ can also spontaneously insert into PPIX. Zn-PPIX could bind to an abundant soluble heme-binding protein, leading to a photoactive protein that could have reduced soluble Fd and replaced flavin-based electron bifurcation. Williamson *et al.* (2011) have discussed the possible role of Zn-tetrapryrroles as functional intermediates in Chl evolution.

The excited triplet state in Zn-PPIX has a half-life roughly 10^6^-fold longer than Mg-Chl excited states, which typically are in the range of a few nanoseconds for the photochemically active singlet species (Björn *et al.*^2009^). A total of 7–15 ms is virtually an eternity for the purpose of catalyzing a light-driven electron transfer reaction, and brings possible activities into the time domain of diffusion rate-limited chemical reactions with other molecules in the cell. This is ample time for an exited state ^3^Zn-PPIX 'cytochrome' to find by diffusion a cytosolic electron acceptor, such as soluble Fd. Fd is not only a ubiquitous protein in anaerobes (Buckel and Thauer 2013), but is also extremely abundant—in a typical anaerobe, Fd has cytosolic concentrations on the order of 80–400 μM (Thamer *et al.*^2003^) (0.2–1 μmol Fd per gram of cytosolic protein, which is typically 400 mg/ml). The redox potential of a photoexcited Zn-PPIX triplet is about –1.6 V (Dixit, Moy and Vanderkooi 1984); for example, the potential of photo-excited triplet Zn-cytochrome *c* (^3^Zn-PPIX(cyt)) at 25°C and pH 7 is –1.7 V (Shen and Kostic 1996). Photoexcited ^3^Zn-PPIX(cyt) is a strong reductant (Shen and Kostic 1996) that readily reduces both plastocyanin and ferricytochrome *b*_5_ (Qin and Kostic 1994). The redox potentials from ^3^Zn(cyt) to Zn(cyt)^+^ (900 mV) and back to Zn(cyt) (800 mV) would fit with Fd reduction, and with reduction of ZnCyt^+^ by a respiratory chain (Shen and Kostic 1996). A protein-bound, photoexcited ^3^Zn-porphyrin, ^3^Zn-PPIX, or a ^3^Zn-PPIX-cytochrome should have more than sufficient driving potential to reduce soluble Fd. These features could constitute the core of a primordial phototrophic Fd reduction pathway (Fig. 2).

A soluble heme-binding protein carrying Zn-PPIX could have become an alternative to H_2_-dependent, flavin-based electron bifurcation and furthermore paved the way to the advent of Chl biosynthesis. Both synthetic Zn-tetrapyrroles and engineered, cytochrome-bound Zn-tetrapyrroles perform light-dependent redox reactions and have been used in the study of artificial photosynthesis (Razeghifard and Wydrzynski 2003; Hay *et al.*^2004^). Synthetic protein-bound Zn-tetrapyroles continue to be of interest in that regard (Cohen-Ofri *et al.*^2011^). Furthermore, Zn-tetrapyrroles also function in nature. Zn-BChl *a* is used in the RC2 of *Acidiphilium rubrum* (Wakao *et al.*^1996^), and Zn-BChl *a*^΄^ apparently forms the special pair of the type-1 RC in *Chloracidobacterium thermophilum* (Tsukatani *et al.*^2012^). Thus, there is physiological relevance of Zn-tetrapyrroles in modern chlorophototrophy, lending weight to the possibility of their involvement in the origin of phototrophy in a heme-containing chemotroph.

## FROM HEME TO CHL: RECRUITMENT FROM EXISTING PATHWAYS

The next questions are how, and why, did the existence of Zn-PPIX lead to a biosynthetic pathway to produce chlorophyllide *a* and ultimately extend to Chl *a*? Zn-PPIX has very strong absorbance in the blue at about 423 nm but only weak absorbance at ∼550 and ∼600 nm. Because the light environment around vents (see the next section) is deficient in blue light, it would have been strongly advantageous, in terms of harnessing available light, to shift the absorbance of the Zn-PPIX into the red/near-IR region of the spectrum. Initially, Mg-chelatase may not have formed the first step in the Chl biosynthetic pathway. Substitution of Zn with Mg would have shifted the chemistry of the excited state from the triplet state for Zn-tetrapyrroles to the singlet state for Mg-tetrapyrroles. This would likely have required that a protein interacting with a chain of electron acceptors already existed to reduce the probability of the back reaction to reduce the oxidized pigment and increase the lifetime of the charge-separated state. However, it really makes little difference when the Mg chelation step evolved because enzymes of Chl biosynthesis tolerate, to differing degrees, variation with respect to the identity of the metal in the tetrapyrrole ring, Zn or Mg (Jaschke *et al.*^2011^). Fundamentally, these enzymes require that the tetrapyrrole coordinates a metal atom, the insertion of which is the function of the chelatase, the first enzyme in the pathway.

Thus, the next enzyme of concern would have been the enzyme magnesium-protoporphyrin IX methyltransferase (BchM/ChlM) to protect the carboxyl group of the propionate side chain at C-13 of the Chl precursor (Gomez Maqueo Chew and Bryant 2007a). This enzyme is a simple O-methyl transferase, a common enzyme activity that is widespread in ribosomal RNA modification and that was present in ancient cells (Weiss *et al.*^2016^). The next enzyme, BchE, which forms the isocyclic ring (ring E of (B)Chls), is an oxygen-independent oxidative ring cyclase. BchE is a radical-SAM enzyme, another very ancient family of proteins (Broderick *et al.*^2014^; Weiss *et al.*^2016^), and it furthermore bears some resemblance to coproporphyrinogen III oxidase, HemN, which removes the carboxyl groups from two propionate side chains of coproporphyrinogen III to form the two vinyl moieties of PPIX (Layer *et al.*^2003^). Protection of the carboxyl group to prevent decarboxylation from occurring would have promoted the ring closure reaction. Another radical SAM enzyme, BciD, probably arose later, but it also has a related activity (Thweatt *et al.*^2017^). BciD oxidizes the C-7 methyl group of BChl *c* to form the C-7 formyl group of BChl *e*, a 4-electron oxidation, by hydroxylating the methyl group twice to form a *geminal*-diol intermediate, which then apparently dehydrates spontaneously to form the formyl group. By analogy in a Zn-based phototrophic system, one component of the BchE reaction might involve radical-based, sequential hydroxylation of the C-13^1^ position to form a *geminal*-diol that dehydrates spontaneously to form the keto group of isocyclic ring E. The product of this reaction, Zn-divinyl-protochlorophyllide, has absorption maxima at 438, 575 and 624 nm (Jaschke *et al.*^2011^), which would improve light absorbance relative to Zn-PPIX in the orange-red region of the visible light spectrum.

**Figure 3.**
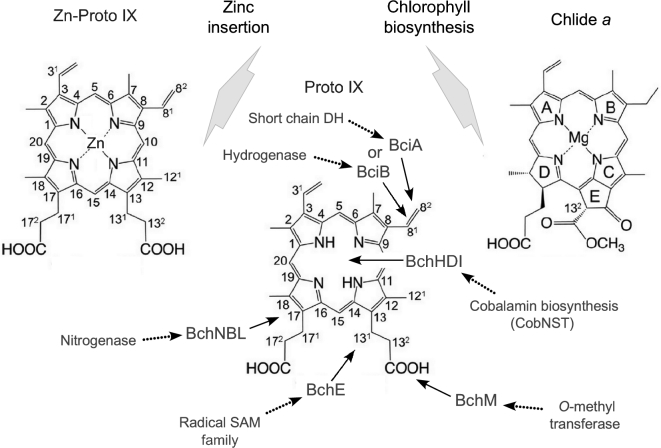
Structural changes and enzymes needed to convert PPIX into chlorophyllide *a*. Modified from Bryant and Liu (2013). PPIX with the changes that generate Zn-Proto IX (left) and chlorophyllide *a* (right). Zn-PPIX is photochemically active and accumulates in Mg-chelatase mutatants of *R. sphaeroides* through a side activity of ferrochelatase (Jaschke *et al.*^2011^). *Chloracidobacterium thermophilum* uses Zn-BChl *a*^΄^ in its RC (Tsukatani *et al.*^2012^). BciD (not shown in the figure) and BchE are both radical SAM enzymes. BciD catalyzes a 4-electron oxidation of the methyl group at C7 to generate a formyl group. A 4-electron oxidation also occurs during the second part of the BchE reaction, part one being the oxidative ring closure (which is possibly similar to coproporphyrinogen III oxidase), part two being the oxidation to produce the keto group of the isocyclic ring E. See the text.

The reduction of the 8-vinyl group (BciA/BciB) may not have been required to produce a highly functional Chl-like molecule, and in any event, it leads to rather small differences in the absorbance spectra of Chls (Gomez Maqueo Chew and Bryant 2007b; Bjorn *et al.*^2009^). BciA and BciB are alternative enzymes and evolutionarily unrelated. As sketched in Figure 3, BciA is a member of the large NADPH dependent short chain dehydrogenase family while BciA is related to F420-reducing hydrogenases typical of methanogens (Sousa *et al*. 2013). Some cyanobacteria, such as *Prochlorococcus* spp., produce divinyl-Chl *a*, which functions equivalently to Chl *a* but has somewhat enhanced absorption of blue light (Chisholm *et al.*^1988^; Goericke and Repeta 1993). At least some cyanobacteria can grow when the gene encoding 8-vinyl-reductase is inactivated (Ito *et al.*^2008^). Finally, a key step in producing a red-absorbing pigment is the reduction of the D-ring double bond to produce the conjugation system of the chlorin ring. A multisubunit enzyme, BchNBL (ChlNBL), which is structurally related to Mo-nitrogenase, would have first catalyzed this reaction (Nomata *et al.*^2006^; Bröcker *et al.*^2010^; Muraki *et al.*^2010^). 8-Vinyl Zn-chlorophyllide *a* has very strong absorption in the red at 664 nm, like Chl *a*, which presumably would have been a desirable property for early light-driven processes (Tamiaki *et al.*^2013^).

Addition of a hydrophobic phytol tail by Chl synthase (ChlG) is all that would then have been required to transfer any phototrophic process(es) that could occur with soluble proteins into the membrane (Gomez Maqueo Chew and Bryant 2007a). This would have had important consequences, because this would have allowed the coupling of redox reactions to energy conservation via ion gradients using pre-existing cytochrome and quinone components. At some point, Zn^2+^ was replaced by Mg^2+^. This may have occurred comparatively late in the origin of phototrophy because the transition from triplet to singlet-based excited state dynamics would have meant that a protein with a series of electron acceptors must have existed to ensure efficient spatial charge separation could occur, and outcompete the rapid back reaction to the ground state that would otherwise have occurred. This may also be why some RCs still apparently employ Zn-BChl *a*^΄^ (Tsukatani *et al.*^2012^). Again, cells would probably have turned to preexisting enzymes. PPIX Mg-chelatase is related to the cobalt chelatase (CobNST) that functions in cobalamin biosynthesis (Debussche *et al.*^1992^), and a pathway to Mg-chelatase would have required only gene duplication and divergence. Thus, all of the enzymes needed to modify Zn-PPIX spontaneously produced from heme biosynthesis to form Chls were probably co-opted from pathways and enzymes already existing in cells (Fig. 3). The one component for which no obvious ancestor has yet been identified is the ancestor of the type-1 RC protein itself. Such a protein probably was a transmembrane, alpha-helical polypeptide, and it is possible that it could have bound a tetrapyrrole (e.g*.* heme) and/or a quinone. Xiong and Bauer (2002a, 2002b) have suggested that cytochrome *b* is a one such candidate.

## ABYSSAL LIGHT, LOW LIGHT, GOOD LIGHT

When biologists (or geochemists) think of the origin of photosynthesis, they often think of harnessing sunlight at the Earth's surface. We are thinking of harvesting light initially from hydrothermal vents (Nisbet, Cann and Van Dover 1995) in the otherwise dark abyss of the ocean floor because, in addition to other reasons to be outlined in this section, that is the site of primary production that was required to support the growth of the heme-containing cells that made the transition to chlorophototrophy (Fig. 2). Hydrothermal vents emit thermal light and visible light.

Thermal light is black body radiation emitted from black smoker types of vents with very hot (>400°C) effluent (van Dover *et al*. 1996; White, Chave and Reynolds 2000; White *et al*. 2002a,b). At such temperatures the emitted light is mainly wavelengths >900 nm, but lesser amounts of light extend down to ∼750 nm, which could be absorbed by chlorosomes (if they existed). Black body light from a >400°C source typically has a vanishingly small component of what is commonly thought of as photosynthetically active radiation (400–700 nm). Because of its low flux in the Chl *a* absorption range, thermal light is not widely discussed as a source of photosynthetically relevant radiation. However, the idea has been alive and well in the literature for over 20 years, starting with the suggestion that the pathway to anoxygenic photosynthesis emerged as a heat- and light-sensing mechanism to guide motile prokaryotes to sources of chemical energy (Nisbet, Cann and Van Dover 1995).

### Low-intensity visible light from hydrothermal vents

Ambient light at hydrothermal vents has a component with wavelengths in the visible range (van Dover *et al.*^1996^; White *et al.*2002a). The first hints for visible light at vents came from studies of a shrimp that inhabits the dark abyss near vents and possesses unusual photoreceptive organs (van Dover *et al.*^1989^). The mechanism(s) generating light in the visible spectrum at vents that exceeds the contribution from thermal light are still unknown; possible sources include sonoluminescence (the collapse of small bubbles) and triboluminescence (light emission from small photoactive crystals) (Tapley, Buettner and Shick 1999; White, Chave and Reynolds 2000). Ambient light flux at vents has been measured directly, and fluxes were reported to be too low to support photosynthetic life, on the order of 10^6^ photons cm^−2^ s^−1^ in the 600–700 nm range (White *et al.*2002a), although higher photon fluxes were also reported, on the order of 10^9^ cm^−2^ s^−1^ in the 700–800 nm range (White *et al.*2002b). Of course, the photon flux at vents decreases with the square of the distance from the source, and can be further reduced by turbidity; at a distance of 2 cm from flange pools at vents along the Juan de Fuca Ridge, photon fluxes on the order of 10^11^ cm^−2^ s^−1^ in the 600–1000 nm range were measured (White *et al.*2002b).

The isolation and cultivation of an obligately photoautotrophic, H_2_S-dependent green sulfur bacterium (GSB) from a hydrothermal vent sample raised the possibility that light emission at hydrothermal vents could indeed be sufficient to support photosynthesis (Beatty *et al.*^2005^). The photon fluxes at vents are on the same order as those observed near the chemocline of the Black Sea, where the flux was reported as 1.8 × 10^11^ photons cm^−2^ s^−1^ (∼3 nmol photons m^−2^ s^−1^). A stable population of a photosynthetic GSB, *Prosthecochloris phaeobacteroides* BS1, lives there at a depth of ∼100 to 110 m and has an estimated *in situ* doubling time of ≥2.8 years (Overmann and Pfennig 1992) or more (Manske *et al.*^2005^; Marschall *et al.*^2010^). The Black Sea GSB uses isorenieratene and BChl *e* to harvest light from the sun (Overmann and Pfennig 1992), which occurs in the blue/green region (*ca*. 450–550 nm) at that depth. The hydrothermal vent GSB uses chlorobactene and BChl *c*, which are better suited for using black body radiation because the *in vivo* BChl *c* absorbance peak is maximal at 750 nm, tailing off around 850 nm (Beatty *et al.*^2005^).

Photon fluxes at vents are about six orders of magnitude lower than surface irradiance for sunlight. A doubling time of 3 years or more might at first seem so slow that one can neglect it, but if we consider modern low-energy environments (Whitman, Coleman and Wiebe 1998), microbiologists are reporting turnover times (not doubling, just carbon turnover whether in the same cell or in a new one) on the order of 1000 years or more (Hoehler and Jorgensen 2013). Our point here is that the photon fluxes observed at vents in the relevant spectral range for chlorophototrophy are much lower than those at the surface, but they could support doubling times that are two to three orders of magnitude faster than those in modern microbial communities in low-energy environments. Thus, considering that microbes do not double in the time domain of hours in the wild, it is possible that the Chl-dependent, photosynthetic lifestyle arose using hydrothermal light. After all, with a doubling time of 3 years, it would take a 1 μm^3^ bacterium only 500 years to produce a cell mass that weighs more than the Earth, given enough substrate.

### Better than sunlight for the origin of phototrophy

In our view, there are reasons to think that a low-light origin of photosynthesis is far more likely than an origin at the surface, where photons arrive a million times faster. The reasons are simple: photooxidation and UV light. Chl absorbs photons and is excited to a highly reducing species (which becomes highly oxidizing if an electron acceptor is nearby). Modern cells must direct those electrons in an orderly flow to acceptors, and obtain reductant at a rate that keeps light-activated Chl from causing harmful oxidations of the cell constituents (Krause and Weis 1991; Demmig-Adams and Adams 1992; Garcia-Medosa *et al.*^2011^). However, most modern Chl-related, high-light damage relates to interactions with O_2_ (Szabo, Bergantino and Giacometti 2005), whereas Chl synthesis arose in the absence of O_2_. Chl triplets can reduce yields, but they do little damage to cells when there is no O_2_ around, and as noted above, triplet states might have initially provided advantages to cells. However, UV light can easily produce the second excited state of Chl (or higher), and can lead to oxidized Chl, which is a very dangerous molecule, one of the strongest oxidants known in biology (Ishikita *et al.*^2005^). Modern cells that use Chl go to great lengths to protect themselves from its oxidative power. At the origin of Chl synthesis, cells would have possessed no mechanisms to protect themselves from oxidants the strength of oxidized Chl because they had never seen such a strong oxidant. Although it has recently been suggested that atmospheric gasses other than ozone might have absorbed some UV radiation during the Archean (Muller *et al.*^2016^), in the absence of an ozone layer, there was certainly a higher flux of photosynthetically active radiation at shallower depths of the water column than at vents on the ocean floor, and sunlight was accompanied by some level of UV radiation, which is not present in hydrothermal light.

Existence near the heavily irradiated, aquatic surface was thus a life-threatening situation for a primitive Chl-containing cell. Moreover, if early primary production was physically linked to H_2_ production via serpentinization at hydrothermal vents, then before the origin of photosynthesis *there was no reductant at the surface from which cells could live*; the UV-irradiated ocean surface harbored neither high local concentrations of a reductant (H_2_) for chemosynthetic primary production to support the growth of the first microbes that invented photosynthesis nor did it harbor high local concentrations of an alternative electron donor (H_2_S) that early photosynthesizers might have harnessed. One could argue that hydrothermal sites in shallow water could have provided the substrates required to support the origin of photosynthesis, but an origin of Chl-based photosynthesis from hydrothermal light seems more likely, because it offers the opportunity of harnessing light energy without the risk of photooxidative damage from UV radiation. In modern oceans, the deep penetration of UV light is attenuated by scattering and absorption due to the presence of living cells and associated chromophoric organic matter (Tedetti and Sempéré 2006). Before the origin of chlorophototrophy, such factors would not have attenuated UV light penetration. Therefore, from the standpoint of a bacterium in its environment, dim light emitted from hydrothermal vents—the only habitat where cells were stably growing on the early Earth—provides the most likely illumination for the origin of photosynthesis.

## TYPE 1 RCs OR TYPE 2 RCs FIRST?

One of the classical questions in the evolution of photosynthesis is which kind of RC came first, type 1 or type 2. The two types of RC are related at the level of structure (Schubert *et al.*^1998^; Allen 2005; Sadekar, Raymond and Blankenship 2006; Blankenship 2010; Cardona, Murray and Rutherford 2015), but with undetectable homology in amino acid sequence alignments. In anoxygenic photosynthesis, type 2 RCs support cyclic electron flow. The electron acceptors of and donors to the RC (quinones and cytochromes and cytochrome-like electron carriers) are supplied by the cell, and an ion gradient is generated that is used for ATP synthesis or reverse electron transport to generate reductants. Anoxygenic type 1 RCs mostly support linear electron flow, and they typically generate Fd_red_ from H_2_S or organic compounds.

If the type 2 RC arose first, then the initial function of chlorophototrophy in the context of increasing primary production was to support the synthesis of ATP and NAD(P)H via reverse electron transport. However, using organic electron donors such as succinate does not increase net primary production. In other words, if a type 2 RC arose first, then the synthesis of NAD(P)H via reverse electron transport, as in the case of photoferrotrophs like *Rps. palustris* TIE1 (Bird, Bonnefoy and Newman 2011), appeared in evolution before the RC1-dependent Fd reduction for CO_2_ fixation, as in the case of the GSB *Chlorobaculum tepidum*. That possibility seems unlikely because it would mean that autotrophs started off dependent on electron bifurcation to synthesize Fd_red_ for CO_2_ fixation, took an evolutionary detour through NAD(P)H and then returned to Fd_red_ with the origin of the type 1 RC.

If the type 1 RC arose first, the initial benefit of chlorophototrophy was to provide access to a new reductant—H_2_S—thereby providing a selective advantage and enabling increased net primary production. To us it seems more likely that photosynthesis started with RC1 than with RC2. Our logic here follows a traditional line of reasoning in photosynthesis evolution, but applies it to a different setting. That is, the traditional logic for the origin of oxygenic photosynthesis from anoxygenic photosynthesis has been that it afforded cells access to a new reductant: H_2_O. By the same reasoning, we suggest that the original function of photosynthesis was to provide access to a reductant other than H_2_, namely H_2_S. H_2_S, like H_2_, is abundant in hydrothermal effluents (Kelley, Baross and Delaney 2002). The initial physiological consequence of phototrophy (and the reason for its evolutionary success) was that it replaced flavin-based electron bifurcation as a mechanism to generate low-potential Fd_red_ for CO_2_ fixation. The first chlorophototrophs made the step into an ecologically new world that was no longer dependent on H_2_. Chl-dependent photosynthesis represented light energy-supported access to a new reductant, H_2_S, in a highly reducing, low-potential redox environment that harbored three essential prerequisites for photosynthesis evolution:

1. a preexisting reductant (H_2_) to support the survival of cells while they were evolving Chl synthesis,
2. access to a new reductant (H_2_S) requiring light energy for oxidation but with a low midpoint potential (HS^−^/S° couple, *E*^o΄^ = −270 mV), and
3. a continuous low light flux that was free of UV photons, providing the first cells that started to accumulate Chl an opportunity to adapt to its presence, rather than suffering damage from UV-induced oxidized Chl as the photobiological situation at shallow depths would have presented.

It is true that anoxygenic chlorophototrophic bacteria can use the type 2 RC for reverse electron transport and photoferrotrophic growth (Bird, Bonnefoy and Newman 2011), yet the main function associated with RC2 today is cyclic electron transport (except in conjunction with PSI as in cyanobacteria). The main function associated with RC1 is linear electron flow from H_2_S to produce Fd_red_. We suggest that it has always been that way, and that the origin of RC1 was thus the first decisive step in the origin of photosynthesis. We do not offer a suggestion for the precursor protein from which the RC1 proteins arose; it is possible that some Chl-lacking, heme-containing protein with structural similarity to RC1 might someday be found, conceivably a cytochrome *b* homolog (Xiong and Bauer 2002a, 2002b); however, the nature of the RC protein precursor is not essential here.

## H_2_S FIRST OR Fe^2+^ FIRST?

Electron donors for anoxygenic photosynthesis include H_2_, H_2_S and Fe^2+^ (Table 1), and certainly all would have been present at a hydrothermal vent in the Archean. H_2_ is the least likely electron donor for the first chlorophototrophs. H_2_-dependent chemotrophs can grow at H_2_ partial pressures as low as 1 Pa (Thauer 2011) using one or more of the three kinds of hydrogenase known—[Fe-Ni], [Fe-Fe] and [Fe] (Shima *et al.*^2008^). Autotrophs that use H_2_ to reduce Fd via electron bifurcation would have had no benefit from photosynthesis. They would have remained dependent on H_2_ but with an additional dependence (on light). That is, they would have had to evolve and employ an energetically expensive machinery (Chl synthesis and RC biogenesis) that only added redundancy to a simpler, preexisting, highly tuned and fully functional chemolithoautotrophic metabolism, without eliminating H_2_ dependence or gaining any net benefit.

For that reason, the suggestion that H_2_ was perhaps the first photosynthetic electron donor (Tice and Lowe 2006) seems unlikely to us. Harnessing H_2_, which the first autotrophs could use without Chl anyway, would not have increased primary production, nor is light required for autotrophic growth on H_2_ (Thauer *et al.*^2008^; Buckel and Thauer 2013; Schuchmann and Müller 2014), which may explain why the acetyl-CoA pathway is not used in conjunction with phototrophy. Phrased another way, if H_2_ is the first electron donor for chlorophototrophs, it provides no benefit to the cell that evolved photosynthesis because the final benefit of the extensive evolutionary investment (Chl biosynthesis, RC origin, integration into the electron transport chain) is the ability to do what cells could do from the outset, namely access H_2_ as a reductant for CO_2_ fixation. In this regard, we note that an RC would affect neither the affinity of a preexisting hydrogenase for H_2_ nor that of a CO_2_ reducing enzyme for its substrates.

We suggest that the first electron donor for anoxygenic photosynthesis was H_2_S (HS^−^), not Fe^2+^, because the evolutionary and electrochemical leap in redox midpoint potential from H_2_ (–414 mV) to access the HS^−^/S° couple (–270 mV) is of far lesser magnitude than that needed to access Fe^2+^ (*E*^o'^ = *ca*. + 150 mV at pH 7). Furthermore, in photoferrotrophs characterized so far, Fe^2+^ oxidation entails cytochromes and high-potential iron-sulfur proteins (HiPIPs) with much more positive midpoint potentials (Bird, Bonnefoy and Newman 2011; Crowe *et al.*^2017^). For example, PioA from the phototrophic iron oxidation operon of *Rps. palustris* TIE1, which uses a type 2 RC, is a decaheme cytochrome, and PioC is a HiPIP (Bose and Newmann 2011); their midpoint potentials reside in the range of +385 to +450 mV (Bird, Bonnefoy and Newman 2011). FoxE in *R. ferrooxidans* SW2 is the iron-oxidizing protein and is a diheme cytochrome, which also has a high midpoint potential in the range +201 to +300 mV at pH 7 to 6 (Saraiva, Newman and Louro 2012). The Cyc2-type cytochromes that photoferrotrophs employ in iron oxidation typically have very high midpoint potentials in the range of +560 mV (Bird, Bonnefoy and Newman 2011), and appear to be the same type of protein that aerobic iron oxidizers, such as *Acidithiobacillus ferrooxidans* (Ishii *et al.*^2015^) or *Mariprofundus ferrooxydans* PV-1 (Barco *et al.*^2015^), use to oxidize Fe^2+^ to Fe^3+^, with O_2_ serving as the terminal acceptor. The same high-potential cytochrome, also called Cyc2_PV-1_ (Barco *et al.*^2015^), occurs in the iron-oxidizing photoferroautotrophic GSB, *Chlorobium phaeoferrooxidans* (Crowe *et al.*^2017^), which uses a type 1 RC. The electrons from Cyc2_PV-1_ in *M. ferrooxydans* PV-1 are thought to be transferred to a soluble periplasmic di-heme cytochrome, called Cyc1_PV-1_ (Barco *et al.*^2015^), which is thought to function similarly (electron transfer) to FoxE in *Rhodobacter* sp. SW2 and PioA in *Rps. palustris* TIE1 (Bird, Bonnefoy and Newman 2011). In the case of the photoferroautotrophic GSB electrons are transferred to a type-1 RC, whereas in photoferroautotrophic *Rps. palustris* TIE1 a type 2 RC performs cyclic electron transfer to energize reverse electron transport, but in both cases high-potential cytochromes are involved.

There is only one isolated report of photoferrotrophic growth of cyanobacteria (Cohen 1984). Siderophilic (iron-loving) cyanobacteria have been isolated from iron-rich environments (for example, *Leptolyngbya* sp. strain JSC-1, also known as *Marsacia ferruginose;* Brown *et al.*^2010^). This organism requires high concentrations of iron for growth and forms iron deposits inside and outside cells; however, convincing evidence for photoferrotrophy in this organism is still lacking. Because cyanobacteria oxidize water and produce oxygen, spontaneous oxidation of Fe^2+^ complicates analyses, and Fe^3+^ is toxic in the presence of oxygen. Given that water is 55 M and Fe^2+^ concentrations are generally in the micromolar range, there would seem to be little selection pressure for cyanobacteria to oxidize iron under most circumstances. Thus, if modern cyanobacteria do oxidize iron, one might expect this process to occur in environments that undergo alternating periods of oxic and anoxic conditions during the diel cycle (e.g*.* hot spring microbial mats (Jensen *et al.*^2011^)).

In RC2-containing photoferrotrophs (for example, *Rhodobacter* or *Rhodopseudomonas* spp.), CO_2_ fixation occurs via the CBB cycle, one of the most recent Fd-independent pathways of CO_2_ fixation to have arisen (Fuchs 2011). Of course, *Rhodobacter* spp. are capable of reducing Fd with the help of RC2 using Rnf (an NADPH:Fd oxidoreductase) that harnesses the photosynthetic transmembrane potential to produce Fd_red_ for nitrogen fixation (Schmehl *et al.*^1993^; Biegel *et al.*^2011^) by reverse electron transport. However, the electrons for Fd reduction in *Rhodobacter* spp. typically come from substrates such as organic acids in RC2-based phototrophy and the process is driven by ATP hydrolysis (Hoffmann *et al.*^2015^).

### Modern photoferrotrophy lacks ancient traits

One could argue that photoferrotrophy involving RC1, as in *C. ferrooxidans*, came before H_2_S oxidation involving RC1, but any strictly anaerobic cell that was in the process of synthesizing Chl in a manner that would not have been suicidal would have been far more likely to access the HS^−^/S° couple (*E*^o'^ = –270 mV), rather than the much more positive Fe^2+^ oxidation step (*E*^o'^ = *ca*. + 150 mV at pH 7), based on what we currently know about electron donors in anoxygenic RCs. Oxidation of either H_2_S or Fe^2+^ is not likely to have been an activity of the RC itself; it probably would have required an enzyme or intermediate cofactor (e.g. cytochrome). For the HS^−^/S° couple, preexisting pathways involved in S^0^ reduction could have been recruited to operate with the same substrate but in reverse. That would not have been an option in the case of the Fe^2+^/Fe^3+^ couple, for lack of environmental Fe^3+^ and hence Fe^3+^ reducers prior to the great oxidation event (GOE).

High-potential cytochromes involved in Fe^2+^ oxidation seem to be more typical of O_2_-respiring bacteria, and thus appear to be derived mechanisms of Fe^2+^ oxidation that might have come into combination with RCs later in evolution, possibly after the appearance of O_2_. We are confronted with the problem that not only is photosynthesis mobile across broad taxonomic boundaries, but also iron oxidation and O_2_ reduction (terminal oxidases) are probably mobile too, making it difficult to identify the directions and the timing of horizontal gene transfers (Castresana *et al.*^1994^; Soo *et al.*^2017^).

The existence of photoferrotrophy in GSB and proteobacteria does not preclude the possibility that other forms of photoferrotrophy might have existed before the appearance of O_2_. These would have entailed theoretical mechanisms of electron entry into the photosynthetic electron chain that are independent of high-potential cytochromes and HiPIPs. Such mechanisms of photoferrotrophy might exist but remain to be discovered. Importantly, we are *not* calling into question the idea that photoferrotrophy per se is a very ancient form of metabolism (Widdel *et al.*^1993^; Ehrenreich and Widdel 1994; Heising *et al.*^1999^). Neither are we questioning the possibility that photoferrotrophy may have been causal to the deposition of the banded iron formations (BIF) (Widdel *et al.*^1993^; Ehrenreich and Widdel 1994; Heising *et al.*^1999^; Kappler *et al.*^2005^). We do note, however, that Crowe *et al.* (2014) pointed out that the photoferrotrophy of GSB may have had a low contribution to BIF deposition under low light conditions, and that Grassineau *et al.* (2006) have pointed out that the sulfur cycle during the Archean was probably very similar to that of today based on sulfur isotope data.

We are simply stating that, from the standpoint of physiology, the nature of electron flow, and the cofactors involved in the forms of photoferrotrophy characterized so far, this metabolism does not look very ancient in any respect (far less ancient than H_2_S-dependent phototrophy in GSB, in particular). Rather, it appears to result from the acquisition of a couple of high-potential cytochromes (and HiPIPs) by horizontal gene transfer, which were incorporated into the metabolism of bacteria that were already able to grow photoautotrophically.

It is also possible that the photoferrotrophs characterized so far are different lineages than those supposedly involved in BIF deposition, even though the underlying chemical process (light-dependent Fe^2+^ oxidation) is the same. These same kinds of basic questions—namely, are the bacteria that perform these processes today direct descendants of the bacterial lineages that performed the processes 3 billion years ago, and are the processes even the same, pervade the literature on the evolution of photosynthesis. They also pervade the literature on Earth history because photosynthesis is so closely tied to geochemical evolution. Because horizontal gene transfer decouples physiology from phylogeny (Martin 2016; Wagner *et al*. 2017), it is important to make sense out of the evolution of photosynthesis in a manner that is not dependent on branching orders in trees (while not completely ignoring circumstances where trees might be relevant and provide insights).

Of course, photothiotrophy requires a sulfide-oxidizing enzyme to access the reductant. The enzymology of sulfide oxidation was probably not the limiting step, however, as there are at least three phylogenetically unrelated isoenzymes that oxidize H_2_S—sulfide:quinone oxidoreductase (SQR), flavocytochrome *c/*sulfide dehydrogenase (FccAB) and a rhodanese-like protein, SoxL (Dahl 2017)—in addition to several unrelated enzymes that can reduce S^0^ to H_2_S, including the NADPH-dependent sulfur reductase Nsr (Bridger *et al.*^2011^) and polysulfide reductase Psr (Jormakka *et al.*^2008^). The existence of such enzymatic diversity indicates that the evolution of H_2_S oxidation systems (or reversing electron flow through pre-existing S^0^-reducing enzymes) is not an evolutionary hurdle per se; the hurdle is the origin of Chl-based, light-dependent oxidation.

## INTERMEZZO

To summarize so far, Chl biosynthesis (from the heme precursor PPIX) was the initial step of photosynthesis evolution. Zn-tetrapyrroles might have played a role as intermediates in Chl origin (Williamson *et al.*^2011^). Chl probably arose in an anaerobic bacterium that possessed cobalamin, cytochromes and quinones. That bacterium was furthermore a heterotroph or facultative heterotroph, and it probably lived near a hydrothermal vent, because that was where electron bifurcation-dependent primary production was occurring. This early evolutionary line could have made the first steps towards chlorophototrophy using hydrothermal light because at that time low-intensity, long-wavelength light presented an opportunity, whereas high-intensity UV light would have been deadly. Most lineages of photoautotrophic bacteria use H_2_S as an electron donor. The first RC probably functioned similarly to the type 1 RC of a GSB, coupling light-dependent H_2_S oxidation to the generation of Fd_red_, replacing the function of flavin-based electron bifurcation in H_2_-dependent chemolithoautotrophs as the fulcrum of primary production and affecting primary production in two significant ways: it allowed primary production to increase, and it released the physical constraint tying primary production to hydrothermal sources of H_2_. Obligately photoautotrophic GSB present at hydrothermal vents today might harbor an ancient kind of low-light phototrophy analogous to that existing at the onset of phototrophy, but need not represent the most ancient chlorophototrophic lineage, because of horizontal gene transfer.

With the ability to harness light in a linear electron transport chain leading from H_2_S to Fd_red_, light limitation would eventually become as important as reductant limitation in such an environment. This would have provided a selective advantage to improvements and modifications of Chl biosynthesis that generated functionally specialized pigments. Perhaps more immediately, it would have conferred advantages to cells that could most efficiently harvest low-intensity light: that is, to develop an antenna like the chlorosome, which is simple in design, unparalleled in light-harvesting efficiency and energetically inexpensive to produce compared to protein-based antenna systems (membrane-intrinsic antenna complexes, or phycobilisomes). Chlorosomes may resemble the ancestral state of light harvesting (Bryant and Liu 2013). Photoferrotrophy, as found in currently characterized proteobacteria and GSB (Table 1), is most plausibly interpreted as the recent horizontal acquisition of cytochromes that can oxidize Fe^2+^ (from bacteria that can oxidize Fe^2+^ using O_2_ as the terminal electron acceptor) by these chlorophototrophs.

## SULFIDE AND A POSSIBLE PATH TO WATER SPLITTING

### A second photosystem, for what?

Evolution in prokaryotes does not proceed under direction, nor does it seek out new solutions; it proceeds via gene duplication, mutation, (re-)combination and horizontal transfer, and it is advanced by natural selection. Once cells had evolved the ability to access H_2_S and light using Chl, standard Darwinian trial-and-error tinkering would have begun to integrate photothiotrophy into the preexisting physiology and genetic composition of the cell (Bauer and Bird 1996; Allen 2005). Photosynthetic life at low light intensities would be a primitive trait in our scenario, and chlorosomes, exceedingly efficient antenna complexes requiring only a few conserved proteins (Bryant and Liu 2013), probably represent one of the earliest forms of light-harvesting antenna complexes. However, the limited and skewed phylogenetic distribution of chlorosomes, their occurrence in combination together with either RC1 or RC2 (Table 1) and the small number of proteins (beyond Chl biosynthesis) required for their biogenesis suggests that they, too, could be subject to horizontal transfer in evolution. Primary production based on the oxidation of H_2_S should have been a stable physiology.

**Figure 4.**
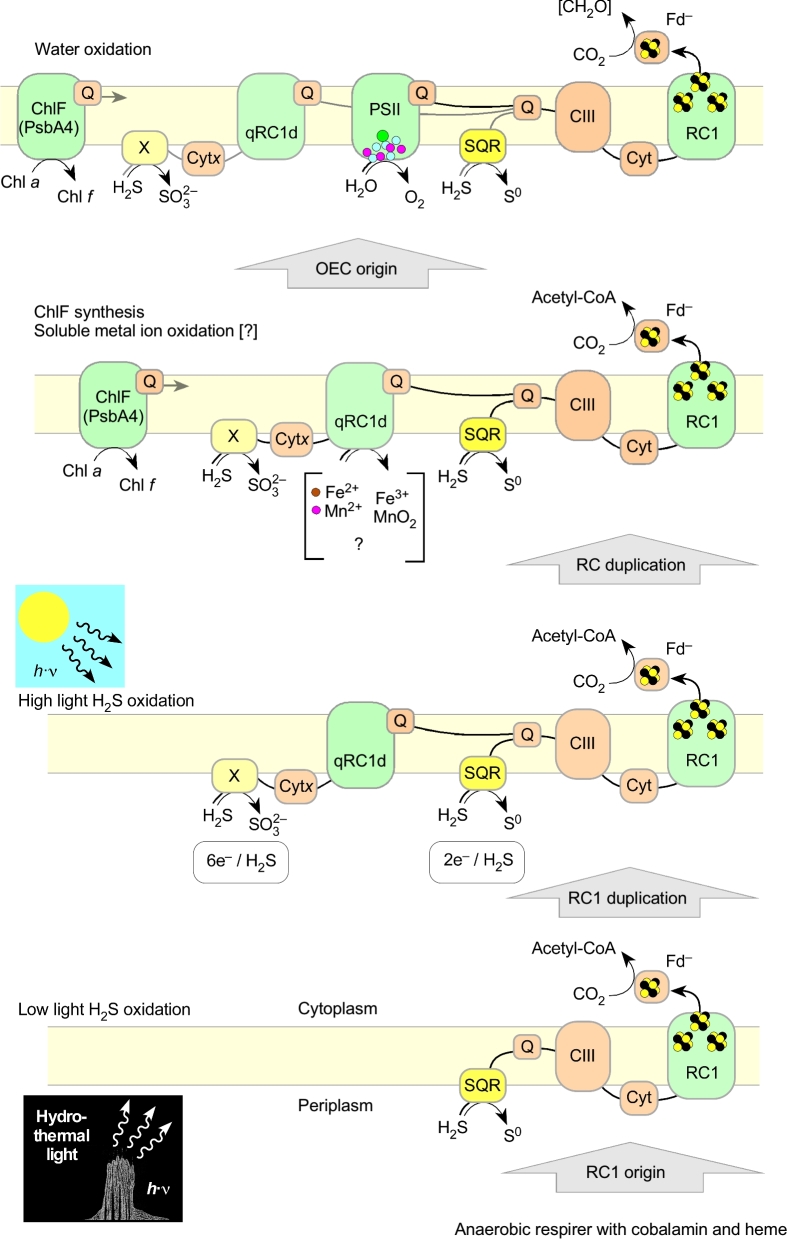
Photothiotrophy in the evolution of two photosystems. SQR: sulfide:quinone oxidoreductase. CIII: complex III-like cytochrome complex. Q: quinone. qRC1d, a quinone-reducing RC1 duplicate. X: Sulfite-generating protein. Cyt *x*: hypothetical carrier (probably a cytochrome). See the text.

There are two main questions about the origin of two types of RCs: how and why? The former is much easier to answer than the latter. The main contours of our proposal are summarized in Fig. 4.

There are two ideas for how two RCs came to be: gene duplication and functional diversification within one organism (Olson and Pierson 1987; Allen 2005), and vertical divergence by means of lineage-specific RC evolution prior to horizontal gene transfer to bring a second RC (RC1? RC2?) into a protocyanobacterium (Hohmann-Mariott and Blankenship 2011; Blankenship 2017). This question is readily answered on the basis of the Chl biosynthesis pathway. The RC proteins of RC1 and RC2 are structurally related but deeply divergent in terms of structure, subunits, pigment binding, amino acid sequences and function (Schubert *et al.*^1998^; Sadekar, Raymond and Blankenship 2006; Gisriel *et al.*^2017^). What they share in common, however, is that neither RC type can undergo any sustained evolution (that is, avoid pseudogenization and loss) without Chl (Sousa *et al.*^2013^). Thus, in the case of lineage diversification and vertical divergence, there should exist, and we should expect to observe, two deeply divergent pathways of Chl biosynthesis in nature: one associated with RC1 (at least sometimes) and one associated (at least sometimes) with RC2. In the case of gene duplication within a single lineage (RC1 yields RC2 or vice versa), Chl biosynthesis should be monomorphic—that is, not existing in two deeply divergent manifestations.

Although not widely acknowledged, this question has already been answered: there is only one pathway for Chl biosynthesis leading from Mg-protoporphyrin IX to chlorophyllide *a* in modern chlorophototrophs. This is strikingly different from the case of heme biosynthesis, for which pathway variations exist (Dailey *et al.*^2017^). Because the available evidence indicates a single pathway, it appears that the two RC types arose in the same genome via gene duplication (Sousa *et al.*^2013^). We note that there are alternative forms for the relatively recently evolved O_2_-dependent and more ancient O_2_-independent isoenzymes—sometimes found within the same genome (Chen, Canniffe and Hunter 2017), and so some extant Chl biosynthetic enzymes arose after the origin of O_2_. How RCs came to exist in the genomes where they are currently found, namely vertical inheritance vs horizontal transfer, is a different question that will be briefly addressed below.

### Two RCs and two entry points for sulfide

We have outlined above reasons why RC1 should be the ancestral RC type. The original function of the product of gene duplication and divergence, RC2, is more difficult to ascertain. In a bacterium that was oxidizing H_2_S with the help of RC1 (and probably SQR), the function of the initial duplication of RC1, RC1d (meaning initially identical in sequence to RC1), would have been to do what it had been doing before the duplication; namely, the RC1d would help to oxidize H_2_S by transferring electrons from cytochromes to Fd. In anoxygenic bacteria, both RC1 and RC2 oxidize cytochromes. RC1 reduces Fd via three [4Fe4S] clusters at the cytosolic (acceptor) side of the protein complex whereas RC2 reduces quinones (Golbeck 1993). Both RC electron-transfer pathways involve the participation of a cytochrome *c*:quinol oxidoreductase, usually similar to Complex III, although *Chloroflexi* and *Acidobacteria* use alternative Complexes III (Bryant *et al*. 2012; Bryant and Liu 2013). We will not deal with possible modifications within the RC, but instead focus on substrate oxidation and product reduction.

Assuming RC1 to be the ancestral state, the evolutionary and functional modifications would occur in its duplicate gene. Loss of FeS clusters and their replacement by a quinone-binding pocket would have led to a quinone-reducing RC1 duplicate, labeled qRC1d in Fig. 4, which retained the ability to oxidize cytochrome, but probably a different cytochrome from that recognized by RC1 (hence labeled Cyt *x* in Fig. 4). This would have generated the capacity for combined linear and cyclic electron transport, and two types of RC that could be differentially expressed to serve ATP synthesis or Fd reduction, as needed by the cell (Allen 2005). This hypothetical intermediate state is almost identical to that suggested by Allen (2005), although our starting point is different. Allen (2005) suggested that from this intermediate state, which he termed a ‘heteronuclear anoxygenic phototroph’ (HAP), there was a loss of differential regulation from the ‘OR’ state to the ‘AND’ state, leading to expression of both RCs simultaneously. Evolutionary exploration led to an RC producing a very strong oxidant, initially oxidized Chl but eventually the tyrosyl radical of RC2 prior to the origin of a Mn-based water-splitting complex. Oxidation of soluble, environmental Fe^2+^ or Mn^2+^ may have represented an intermediate ancestral state from which the water-splitting complex arose. There is geochemical evidence to suggest that just prior to the GOE, soluble Mn^2+^ oxidation occurred in marine environments (Johnson *et al.*^2013^). The GOE is widely interpreted as marking the global appearance of O_2_ in the geochemical record (Fischer, Hemp and Johnson 2016).

### Sulfide oxidation to sulfite and thiosulfate

We suggest that the initial physiological function of an RC1d module was sulfide oxidation to sulfite and thiosulfate, which is based on the following three observations: (i) many cyanobacteria oxidize H_2_S in a light-dependent manner to produce S_2_O_3_^2–^ (thiosulfate) rather than S^±0^, indicating that SQR is not the sulfide-oxidizing enzyme (de Wit and van Gemerden 1987; Rabenstein, Rethmeier and Fischer 1995); (ii) light-dependent H_2_S oxidation to thiosulfate in the cyanobacterium *Microcoleus chthonoplastes* was initially reported to be insensitive to DCMU (3-(3,4-dichlorophenyl)-1,1-dimethylurea)), an inhibitor of PSII-dependent quinone reduction (de Wit and van Gemerden 1987), but in a later study on the same organism, thiosulfate production was reported to be inhibited by DCMU (Rabenstein, Rethmeier and Fischer 1995); (iii) highly divergent copies of *psbA*, which encode products that lack the residues crucial for the binding of the Mn_4_CaO_5_ cluster in the oxygen-evolving complex (OEC), exist in many cyanobacterial genomes (Murray 2012). One of these was recently shown to perform a light-dependent oxidation in Chl biosynthesis to generate Chl *f*, and was hence renamed ChlF to reflect that function (Ho *et al.*^2016^). ChlF is a new type of homodimeric, DCMU-sensitive photo-oxidoreductase that uses light to perform a four-electron oxidation of Chl *a* or chlorophyllide *a* (Ho *et al.*^2016^; G. Shen and D. A. B., unpublished). In phylogenetic trees, ChlF is an early diverging member of the *psbA* superfamily that retains most of the functional features of the RC protein PsbA of PSII, except for the ability to bind the Mn_4_CaO_5_ cluster (Murray 2012; Cardona, Murray and Rutherford 2015; Ho *et al.*^2016^; Cardona *et al.*^2017^).

PSII itself is an unlikely candidate for H_2_S oxidation because it is highly specialized and not known to function with reductants other than H_2_O or H_2_O_2_. Furthermore, PSII activity is inhibited by sulfide, although some cyanobacterial strains show much greater tolerance to phototrophic sulfide inhibition than others (Miller and Bebout 2004). As depicted in Fig. 4, the main difference between our intermediate and Allen's HAP is that the new RC1-derived, sulfide-oxidizing module would allow cells to introduce electrons from H_2_S at different electron yields into a linear electron transport chain via two low-potential sites: qRC1d (H_2_S to S_2_O_3_^2–^, E^o'^ = –193 mV, a six-electron reaction) and/or RC1 (H_2_S to S^±0^, E^o'^ = –270 mV, a two-electron reaction). This also constitutes a difference from the model of Klatt *et al*. (2015), who proposed a single site of sulfide entry to the electron transport chain via SQR. In a recent study of *Geitlerinema* sp. PCC 9228 (formerly *Oscillatoria limnetica* Solar Lake), Grim and Dick (2016) noted that several cyanobacteria, including *Geitlerinema* sp., possess an additional copy of SQR and that these are sometimes located in the genomic proximity of divergent *psbA* gene duplicates.

The benefit for a bacterium with the configuration we propose (i.e*.* two options for oxidizing the same substrate) is evident. The oxidation of H_2_S to SO_3_^2–^ via qRC1d would introduce six electrons per H_2_S into the electron transport chain (Brune 1989), whereas the oxidation of H_2_S to S^0^ via RC1 introduces only two electrons per H_2_S, providing a simple means via differential gene expression of RC1 and qRC1d to modulate the light-dependent reduction state of the quinone pool with respect to the physiological needs of the cell—either at constant light intensity and constant H_2_S concentrations, or in response to changing light and/or H_2_S availability. Obtaining six electrons from H_2_S for carbon reduction and ATP synthesis would obviously be advantageous over the SQR reaction in environments where H_2_S concentration decreased, or when more electrons were needed (for example, during N_2_ fixation). This aspect of our proposal (differential gene regulation to maintain redox balance in the bioenergetic membrane) is virtually identical to that of Allen (2005), but our substrates and gene expression regimens are different. Our dual sulfide-oxidizing intermediate (DSO) would be able to generate different quinone reduction states from the same low-potential substrate, H_2_S, by regulating RC gene expression, whereby the ultimate fate of the DSO and HAP (Allen 2005) hypothetical intermediates—further evolution resulting in water oxidation—is the same.

We offer no specific mechanistic proposal for how light-dependent H_2_S oxidation to sulfite or thiosulfate by protein X in Fig. 4 might occur—whether it entails one electron to two electron-transducing cofactors such as a flavin or quinone, or whether it might involve sulfur radicals. There are many thiyl radicals in biology (Buckel and Golding 2006), including thiyl to tyrosyl transfers as in the reaction mechanism of ribonucleotide reductase (Buckel and Golding 2012). However, the first step in H_2_S oxidation to sulfur or polysulfide is typically a two-electron reaction (Luther 2010; Luther *et al.*^2011^), suggesting that in both modern cyanobacteria and the phototrophic thiosulfate-generating reactions of ancient protocyanobacteria, X might catalyze a two-electron reaction. Nothing is yet known biochemically about how cyanobacteria oxidize sulfide to thiosulfate via sulfite as originally reported by de Wit and van Gemerden (1987) and confirmed by Rabenstein, Rethmeier and Fischer (1995). In particular, the sulfite/thiosulfate-generating enzyme(s) has not been identified. It is possible that thiosulfate results from a spontaneous or enzymatic reaction of a sulfite intermediate with the substrate, sulfide. Protein-bound trisulfides have recently been characterized as intermediates in bacterial sulfite reduction (Santos *et al.*^2015^). In Fig. 4, we designate the so far uncharacterized sulfite-producing enzyme as X and the corresponding intermediate carrier, probably a cytochrome, as Cyt *x*.

### Donors more redox positive than sulfide

Further modification of the periplasmic, oxidizing side of qRC1d to accommodate oxidation of other substrates, such as soluble Mn^2+^, would have given rise to novel and different anoxygenic phototrophic activities as possible intermediates *en route* to water oxidation (Dismukes *et al.*^2001^; Allen and Martin 2007; Johnson *et al.*^2013^; Fischer, Hemp and Johnson 2016). Whether protocyanobacteria could have oxidized soluble Fe^2+^ has not been discussed. In light of the single isolated report of photoferrotrophic cyanobacterial growth (Cohen 1984), and given 2.4 billion years of global O_2_ and low concentrations of Fe^2+^, it is possible that cyanobacteria have lost this trait altogether, that they never had it or that it has not been studied in enough detail. However, with oxygenic photosynthesis in place and energy to burn, so to speak, the transition from rTCA-based autotrophy to the energetically expensive and inefficient CBB cycle became 'affordable’.

The origin of the OEC marks two important transitions. First, it would mark the end of reductant limitation for marine primary production (under the premise that photothiotrophy was quantitatively more significant than photoferrotrophy during the Archean). Second, it marks the endpoint of the evolutionary inference: an oxygenic photosynthetic bacterium possessing two photosystems linked in series, one capable of water oxidation (PSII) feeding electrons into a linear chain to RC1 (PSI) that generates reduced Fd (Fig. 4, top panel). In such an organism, the use of H_2_S as reductant becomes almost irrelevant, useful only under specialized environmental conditions, but essential under conditions of high H_2_S for reasons of PSII inhibition. Occasional essentiality (i.e. a conditionally advantageous capability) would explain why the ancestral trait (two kinds of sulfide oxidation differing in their electron yield) may have been retained in some modern generalist cyanobacterial lineages. By physiological definition (having PSI and PSII, and producing O_2_), such an organism would be a cyanobacterium (or a quasi-cyanobacterium), but it is not essential here whether the organism would branch basal to or within modern, chlorophototrophic cyanobacteria (sometimes also called *Oxyphotobacteria*), nor is it crucial when the bacterium evolved water oxidation capability, provided that it occurred prior to the GOE *ca*. 2.4 billion years ago and—a condition imposed by our present inference—provided that it occurred after the origin of photothiotrophy in the cyanobacterial lineage. The timing, ecological consequences and some possible geochemical consequences of a DSO are briefly considered in the next section.

## PHOTOTHIOTROPHY: UNDERESTIMATED IN EARTH HISTORY

**Figure 5.**
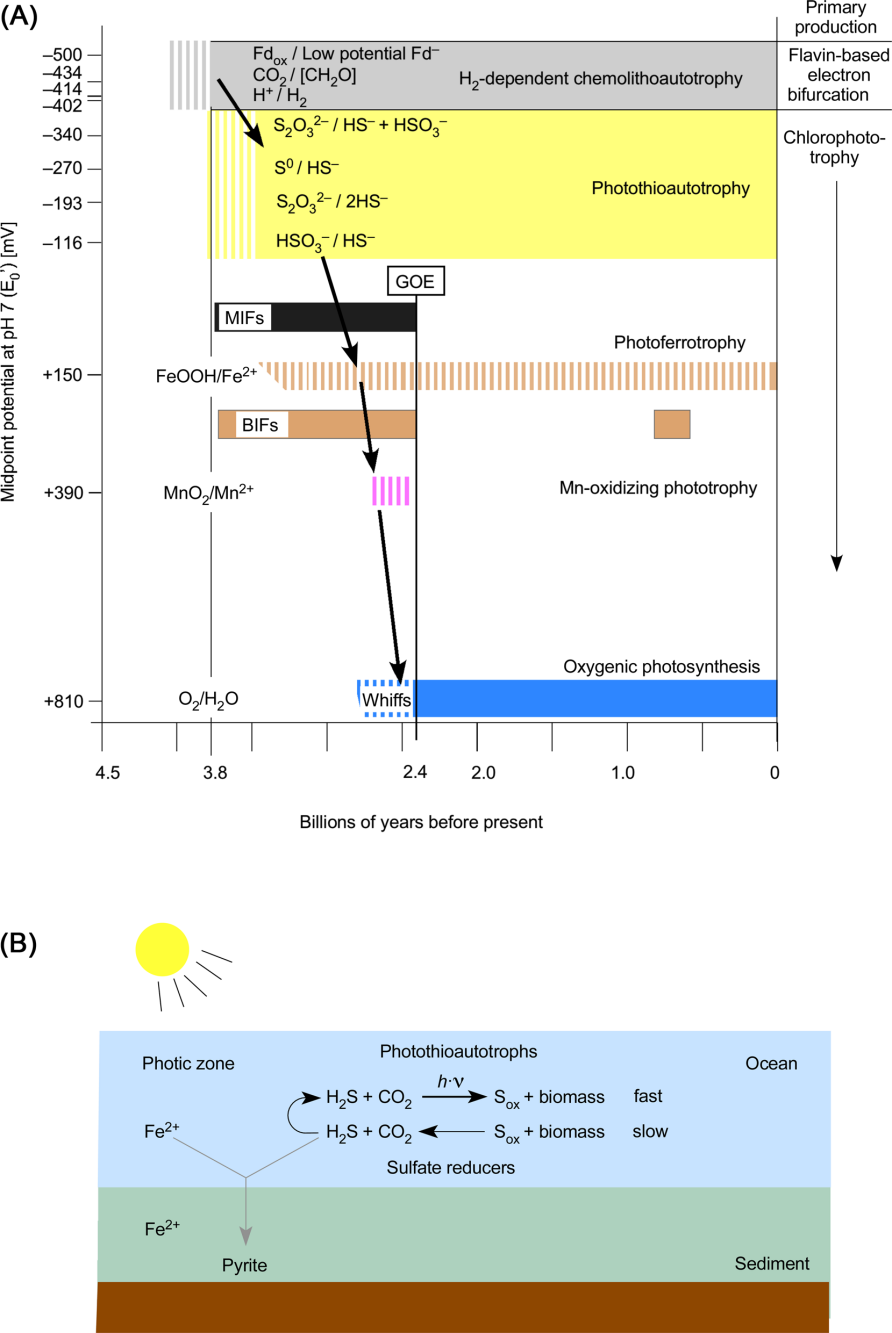
The possible role of photothiotrophy in Earth's history. (**A**) Redox potentials of reductants for primary production vs time. Midpoint potentials are taken from sources listed in the legend of Fig. 1. GOE: great oxidation event. MIFs: mass-independent sulfur fractionations (a proxy for O_2_ absence or presence under 10^−5^ present atmospheric levels in the atmosphere). Mass-dependent sulfur isotope effects are also pronounced prior to the GOE (not shown, see the text). Whiffs indicate the presence of oxidants in the water column or slight O_2_ presence prior to the GOE. BIFs: banded iron formations. The column ‘primary production’ at right underscores the point that before the origin of chlorophototrophy, primary production was H_2_-dependent and mechanistically dependent on flavin-based electron bifurcation (indicated by gray shading). The figure suggests that photothiotrophy preceded photoferrotrophy and water splitting in evolution. (**B**) A possible photothiotrophic sulfur cycle in Archean oceans before the GOE. See the text.

When we turn to the literature on the role of photosynthesis in Earth's geochemical history, three things stand out: BIFs (banded-iron formations), MIFs (mass-independent sulfur fractionations) and, with rare exceptions (for example, Allen 2005), the paucity of attention given to the possibility that photothiotrophy played a major role in Earth's geochemical history prior to the GOE. Johnston *et al.* (2009) proposed an important role for cyanobacterial photothiotrophy, but long after the GOE. Recalling that all modern phyla harboring chlorophotoautotrophs have members that oxidize sulfide (Table 1), and that Allen (2016) recently proposed an origin of Archean stromatolites involving the growth of protocyanobacteria that oxidized H_2_S at RC1—a suggestion that is fully compatible with our present considerations—our thoughts on the possible role of photothiotrophy in Earth's history, summarized in Fig. 5, are as follows.

The salient Earth history observations as taken from three recent reviews (Arndt and Nisbet 2012; Lyons, Reinhard and Planavsky 2014; Fischer, Hemp and Johnson 2016) are fairly straightforward and consistent. Prior to the GOE 2.4 billion (Ga) years ago, there was basically no O_2_ in the atmosphere, as indicated by the presence of MIF. The MIFs disappear simultaneously with changes in other geochemical markers for the presence of oxygen at 2.4 Ga, indicating the presence of a strong oxidant, almost certainly O_2_, in the atmosphere. The BIFs are abundant before the GOE and rare after the GOE. The source of the BIFs is unresolved.

Photoferrotrophy has been widely discussed as a possible biological mechanism to generate BIFs (Camacho *et al.*^2017^), but the numbers observed for the activity of modern photoferrotrophs do not add up in a way that provides a compelling case for their role in BIF formation (Crowe *et al.*^2014^). Lyons, Reinhard and Planavsky (2014) went a step further and suggested that H_2_O was the source of electrons for primary production prior to the GOE, while Fischer, Hemp and Johnson (2016) pointed out that anoxygenic photosynthesis with inorganic electron donors other than H_2_O, and not excluding reduced sulfur species, could account for the same observations (organic-rich shales, high primary production) prior to the GOE.

We suggest the possibility of a quantitatively significant role for photothiotrophy in evolution long before the GOE. Our proposal could be integrated into an Earth history context as follows (see Fig. 5a). H_2_S-based photosynthesis is the ancestral form of anoxygenic photosynthesis, and hence would be ancient, consistent with earlier proposals (Allen 2005). At least some members of all modern photosynthetic phyla (i.e*.* those that fix CO_2_) are capable of using sulfide as an electron donor: *Cyanobacteria, Chloroflexi, Proteobacteria* and *Chlorobi* (Table 1). H_2_S would be the most likely source of high primary production pre-GOE. The duplication of RC1, leading to RC2, produced a highly specialized bacterium with a high affinity for H_2_S and other reduced sulfur species in ocean waters where light was not limiting. The respiratory action of heterotrophic sulfur, sulfite and sulfate reducers converted oxidized sulfur to the reduced state (H_2_S), but high light intensity and dense protocyanobacterial growth kept H_2_S concentrations in the photic zone low, and maintained sulfur in the oxidized state (Fig. 4b). This assumes that neither light nor CO_2_ was limiting for primary production, but that once again in Earth history the reductant for primary production—this time H_2_S instead of H_2_—was limiting. Oxidized S species from photothioautotrophic growth were reduced to H_2_S by sulfate reducers, but at a rate slower than photooxidation. S was cycled like an environmental cofactor, without H_2_S being removed from the photic zone as pyrite, FeS_2_, because H_2_S was oxidized by a light-driven system faster than it was produced by sulfate reducers (Fig. 5B).

Sulfur limitation (arising from variations in its environmental availability) in an Archean photothiotrophic ocean could have generated mass-dependent fractionation of sulfur isotopes prior to the GOE, which is observed (Johnston 2011). With the advent of water oxidation, MIFs would end for the conventional reason: because of the accumulation of atmospheric O_2_. With the replacement of H_2_S with H_2_O as the main reductant for primary production, S would no longer have been limiting for primary production, which should have influenced isotope fractionation effects for S species around the time of the GOE. Moreover, with the origin of water oxidation at the OEC, reductant would no longer have been limiting for primary production. That would have caused a substantial shift in ecosystem function and elemental cycling.

In the geochemical literature, photothiotrophy is sometimes considered to be a process that evolved late, after the origin of oxygenic photosynthesis (Johnston *et al.*^2009^; Johnston 2011) or that was quantitatively of insufficient significance to have produced geological effects (Lyons, Reinhard and Planavsky 2014). Alternatively, it is just not on the map at all, with the effects of MIFs and non-biological processes standing in the foreground (Ueno 2014). Other views mention H_2_S (Klatt *et al*. 2015; Fischer, Hemp and Johnson 2016) but devote little attention to the role of H_2_S as an electron donor in the evolution of photosynthesis. Like Allen (2005, 2016), we see an important role for photothiotrophy in the evolution of photosynthesis and in Earth's history.

Direct evidence for the workings of anoxygenic photosynthesis prior to the GOE is scarce. As Arndt and Nisbet (2012) point out, Frances Westall has reported the characterization of a 3.3 billion-year-old microbial biofilm from an air-exposed setting that was probably performing anoxygenic photosynthesis and may have contained sulfate reducers (Westall *et al.*^2006^, ^2011^). What kind of photosynthesis was supporting that biofilm? The simplest interpretation is that it was anoxygenic photosynthesis (Arndt and Nisbet 2012), as opposed to oxygenic photosynthesis (Lyons, Reinhard and Planavsky 2014), and a likely possibility is that it was photothiotrophy. Of the electron donors used by chlorophototrophs, H_2_S is the most common (Table 1), and it might also be the most ancient. The experiments on photothiotrophs showing relatively small S isotope fractionation effects in laboratory studies have not yet been applied to photothiotrophic cyanobacteria (Johnston 2011). In particular, it will be important to determine whether there is a significant isotopic signature difference between cyanobacteria that oxidize sulfide to sulfur (or polysulfide) and those that produce thiosulfate (see above).

### Whiffs of oxidant

An Archean photothiotrophic ocean would have been stratified in terms of redox chemistry, not due to an absence of mixing but rather due to the bacterial utilization of sunlight. In the geochemical literature, there are phenomena called ‘whiffs’ (Anbar *et al.*^2007^; Knoll, Bergmann and Strauss 2016). Whiffs designate rock formations that were deposited before the GOE and that harbor evidence for the existence of oxidants in the marine water column (Lyons, Reinhard and Planavsky 2014; Fischer, Hemp and Johnson 2016). The causes of whiffs are debated. Some authors interpret them by as evidence for the existence of O_2_ pre-GOE, but at face value they simply indicate the existence of oxidants in the water column. If the Archean oceans were inhabited by bacteria that could perform light-dependent oxidation of sulfide to produce thiosulfate, such activities could be interpreted as evidence for a strong oxidant. Photooxidized Chls are indeed strong oxidants. In PSII, the oxidized special pair of Chls oxidizes tyrosine Z (TyrZ), generating a tyrosyl radical, which is also a very strong oxidant. TyrZ resides close enough to the Mn_4_CaO_5_ water-splitting, OEC to oxidize it (Umena *et al.*^2011^; Shen 2015; Barber 2016).

Prior to the origin of the OEC, the strong oxidant underpinning ‘whiffs’ prior to the GOE could have been a cellular protein-bound oxidant, a Chl cation (P^+^) or a TyrZ-like radical, but not O_2_ itself. In other words, ‘whiffs’ of oxygen prior to the GOE might reflect the existence of a prevalent biological oxidant (RC2) in the photic zone that possessed the midpoint potential to oxidize an OEC, and hence was capable of oxidizing dissolved metals such as Mn^2+^ (Dismukes *et al.*^2001^; Allen and Martin 2007; Johnson *et al.*^2013^; Fischer, Hemp and Johnson 2016), and possibly Fe^2+^, but operated before the OEC had fully evolved. Again, we do not call into question the existence of photoferrotrophy in BIF formation (Widdel *et al.*^1993^; Bird, Bonnefoy and Newman 2011; Camacho *et al.*^2017^) prior to the GOE, or the role of photosynthetic Mn^+2^ oxidation prior to the GOE (Johnson *et al.*^2013^). However, the question of which organisms might have been responsible is open, and we note that the strong oxidant in RC2 of a photothiotroph bearing two types of RCs (a proto-cyanobacterium) could have oxidized other substrates: Mn^+2^ for example, as does an engineered RC2 of *R. sphaeroides* (Allen *et al.*^2012^), and conceivably Fe^2+^. The observation that cyanobacteria oxidize Mn^2+^ in the process of OEC assembly is also relevant in this context (Tamura and Cheniae 1987).

Today, marine primary production is not limited by reductant but nutrients such as iron are limiting. Concerning geochemical models of Earth history, if photoferrotrophy was the predominant form of primary production prior to the GOE, reductant (Fe^2+^) would not have been limiting (although phototrophic biomass might have been), whereas if photothiotrophy was the predominant form of primary production prior to the GOE, reductant (H_2_S) would probably have been limiting until the origin of water splitting. Fitting of geochemical data points to mathematical models (Johnston *et al.*^2009^; Lyons, Reinhard and Planavsky 2014) together with measurements of cyanobacterial S isotope discrimination during photothiotrophic growth might help to discern between these alternatives.

## PHYLOGENETIC DISTRIBUTION OF RCs

Finally, we return to the question of how RC1 and RC2 came to be distributed among modern Chl-producing bacteria. No modern treatment of RC evolution can realistically avoid the problem of horizontal gene transfer; thus, the questions descend to how much and in which direction. An important thing to keep in mind about RC evolution is not which lineages have RCs, but which lineages lack them. Sousa *et al.* (2013) provided one overview of that pattern, but the best rendering of phylogenetic photosystem distribution published to date (to our knowledge) is that in fig. 5 of Fischer, Hemp and Johnson (2016), to which the reader is referred rather than duplicating it here. Our proposal for the distribution of RCs among modern chlorophototrophic lineages has precedent based on bioinformatic surveys (Mulkidjanian *et al.*^2006^), yet differs from previous views in physiological context (photothiotrophy) and in implications: we suggest that RC1 and RC2 arose within the lineage leading to cyanobacteria (one can generically designate members of this lineage as protocyanobacteria), and that those RCs have been vertically inherited in that lineage since the advent of Chl biosynthesis. This suggestion carries a few corollaries, some novel, some not.

### Origin of chlorophototrophy in cyanobacteria

As the first corollary, it would mean that the origin of chlorophototrophy occurred in cyanobacteria and after the separation of the ancestral protocyanobacterial lineage from other bacteria. Here, critics might point to new phylogenies based on ribosomal proteins that were interpreted as evidence for a 'merger hypothesis', that is, that two RCs were transferred into a non-photosynthetic cyanobacterial ancestor to give rise to the lineage (Mathis 1990; Xiong, Inoue and Bauer 1998; Shih *et al.*^2017^; Blankenship 2017; Soo *et al.*^2017^).

Looking at the issue openly, both before and after the publication by Soo *et al.* (2017), there are no bacterial lineages known that branch immediately ancestral to cyanobacteria and that possess only one RC or some other 'primitive' form of photosynthesis. In our view, designating new lineages such as *Melainabacteria* and *Sericytochromatia* and referring to them as ‘cyanobacteria’, is presently unsubstantiated. By their own analysis, Soo *et al.* (2017) concluded that extensive gene loss later occurred in respiring ancestors of gut-residing fermentative melainabacteria, and there is no evidence that loss of photosynthetic capability did not occur in the protocyanobacterial ancestors of these organisms prior to or at the time of the GOE. Hence, it is not evident how the tree of Soo *et al.* (2017), even if its depiction of ribosome lineage relationships holds up over the coming years, directly bears upon the origin and evolution of either Chl or RCs, both of which certainly evolve independently of the ribosome because of horizontal gene transfer. Soo *et al.* (2017) investigated the evolution of the ribosome as a proxy for evolution of the rest of the genome, whereas we are primarily concerned with the origin of the segments of the genome that specify Chl, RCs and autotrophy. The evolution of Chl and RCs is inextricably linked because RCs cannot undergo sustained evolution in the absence of Chl. In contrast, there is no functional linkage between the evolution of ribosomes on the one hand and Chl and RCs on the other. The evolution of ribosomes and chlorophototrophy need not coincide.

Chlorophototrophy did evolve somehow and somewhere, and we suggest that it occurred in the stem lineage of cyanobacteria. Furthermore, as Sousa *et al.* (2013) have pointed out, and as we stress again here, the 'merger' hypothesis for the presence of two RC types in cyanobacteria (Blankenship 2017; Soo *et al.*^2017^) is unlikely to be correct in any case because it predicts the existence of a deep and ancient dichotomy in Chl biosysnthetic pathways leading to the common precursor, chlorophyllide *a*. Such a dichotomy is *not* observed among chlorophototrophic lineages; Chl biosynthesis entails one ancestral pathway, combined in some genomes with the evolutionarily recent O_2_-dependent AcsF alternative to the radical SAM-dependent ring closure reaction catalyzed by BchE (Sousa *et al.*^2013^; Chen, Canniffe and Hunter 2017) (Fig. 3).

Note that nowhere in this discussion have we suggested a timeline for the origin of Chl. We simply suggest that it may have arisen from Zn-PPIX (Figs 2 and 3), that its origin was required for primary production that was not tied to geochemical H_2_ at hydrothermal vents, and that it arose before the GOE. In line with this, Bryant and Liu (2013) pointed out that trees based on (B)Chl biosynthesis suggest the divergence of three lineages that are nearly equally deep—*Chlorobi-Chloroflexi*, *Heliobacteriaceae*-*Cyanobacteria* and *Acidobacteria-Proteobacteria*—each of which contain members with either or both a type 1 and a type 2 RC. One could argue that the duplication leading to heterodimeric PSII, which occurred independently from that leading to PufLM-type 2 RCs, also occurred independently from that of a common RC1-type ancestor, in which (B)Chl biosynthesis already existed.

### Chl *a* first because it arose that way

As the second corollary, an origin of chlorophototrophy in a cyanobacterial progenitor would mean that Chl *a*, which is prevalent in cyanobacteria and is the product of the shortest Chl biosynthetic route (Gomez Maqueo Chew and Bryant 2007a), is also the ancestral Chl, which fits exactly with the Granick hypothesis (Granick 1965) that the evolution of (at least Chl biosynthesis) pathways may recapitulate evolution.

### Cyanobacteria are ancient (but not primordial)

Third, it would mean that the cyanobacteria descend from the most ancient Chl-bearing lineage, without placing a date on their age. This would mean in turn that cyanobacteria are a very ancient line whose origin greatly precedes the GOE, as some interpretations of the fossil record would suggest (Golubic and Lee 1999; Schopf 2012; Knoll, Bergmann and Strauss 2016). This is also very close to what Olson and Pierson (1987) suggested almost 30 years ago, although they had the *Heliobacteriaceae* (*Firmicutes*) lineage branching off before the duplication that gave rise to RCII. Twenty-nine years later, the 'lone ranger' status of *Heliobacterium* and related organisms as the sole chlorophototrophs in the clostridial lineage (Fischer, Hemp and Johnson 2016) might be better explained as horizontal acquisition instead of differential loss among all other *Firmicutes*. This would also fit with the absence of autotrophy in *Heliobacteriaceae* (Table 1), and would not contradict the ancestral features preserved in their RC structure (Gisriel *et al.*^2017^), provided that the transfer was ancient. From the first sections of this paper, it should be clear that we are not saying that the cyanobacterial lineage represents a primordial form of bacteria, we are simply suggesting that it represents the first lineage with chlorophototrophy.

### The export hypothesis

**Figure 6.**
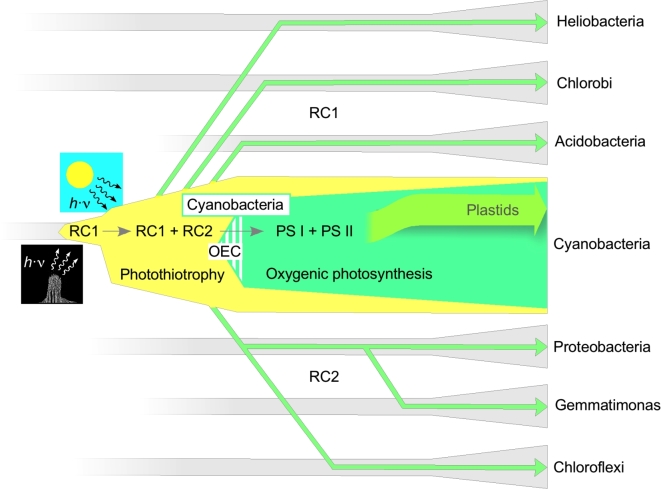
Export hypothesis for the distribution of RCs among oxygenic and anoxygenic chlorophototrophs. Ribosomal lineages are indicated with gray lines; transfer of RCs into recipient lineages are indicated with green arrows. OEC: oxygen evolving complex. The origin of plastids (Sanchez-Barraclado *et al.*^2017^), where cyanobacteria donated both RC1 and RC2 plus the genes for autotrophy to eukaryotes (Ku *et al.*^2015^), is symbolically indicated as a wide arrow branching within the cyanobacterial radiation. The figure makes no statement about the timing of export transfer events relative to the origin of the OEC (probably at or near the time of the GOE) or relative to other transfers. Assuming that a cyanobacterial progenitor invented chlorophototrophy, and that geochemical evidence for ancient phototrophy at around 3.2 to 3.4 Ga before present represents photothiotrophy, then photothiotrophy would trace to that age. The photothiotrophic cyanobacterial lineage existent prior to the origin of the OEC corresponds to protocyanobacteria in our terminology here. Note that we indicate no ribosomal lineage branching patterns at all (except plastids), including *Melainabacteria* or *Sericytochromatia* relative to cyanobacteria, because ancient lineage relationships and branching patterns inevitably change as new phylogenetic methods are employed and as new lineages become known (Williams *et al.*^2013^). Symbols for hydrothermal light at photosynthesis and RC1 origin and sunlight at RC2 origin are indicated. See the text.

Fourth, because none of the anoxygenic photosynthetic lineages branch from within the cyanobacteria, it would mean that *all* anoxygenic chlorophotosynthetic lineages acquired their RCs via horizontal gene transfer ultimately from a cyanobacterial progenitor, although sometimes the transfers might have occurred through intermediate lineages as in the case of *Gemmatimonas phototrophica* (Zeng *et al.*^2014^). This proposal, which we call the 'export' hypothesis (Fig. 6), is precisely the converse of the 'merger' hypothesis (Blankenship 2017); hence, it might not sit well with some proponents of the merger hypothesis, but it would explain a lot. It would directly explain why Chl synthesis undergoes variation and modification emanating from chlorophyllide *a* (requiring only esterification to produce Chl *a*) in anoxygenic photosynthetic lineages. It would also directly explain why RCs are found in combination with different CO_2_ fixation pathways: namely, via RC and (B)Chl operon transfer *without* cotransfer of the *cbb* (CBB cycle) operon; as well as RC + Chl transfer *with* cotransfer of the CBB cycle operon, but involving variation in the latter by replacement of aldolase, RubisCO, bisphosphatases, phosphoribulokinase and GAPDH types (Table 1).

Of course, RC transfer without Chl biosynthesis transfer cannot result in fixation of chlorophototrophic physiology, whereas RC + Chl transfer can give rise to photoheterotrophic lineages. It would also directly explain why so few bacterial lineages, among the countless lineages in nature, are chlorophototrophic, and those that are tend to occur as recent (tip) lineages in phylogenetic trees, rather than branching near the root in their respective groups (see fig. 5 of Fischer, Hemp and Johnson 2016). Acquisition of traits associated with oxygen utilization (a couple of Chl biosynthesis enzymes, terminal oxidases, iron oxidation) in some anoxygenic photosynthetic lineages can be attributed to horizontal gene transfers that occurred post-GOE (Table 1). Shih, Ward and Fischer (2017) suggested that *Chloroflexus* might have acquired its phototrophic ability via horizontal gene transfer, compatible with earlier transfer proposals (Mulkidijanian *et al.*^2006^) and with our present proposal, which, unlike these prior proposals, is not based on phylogenetic trees. Instead our proposal takes into account *Roseobacter* plasmids that carry phototrophic genes (Petersen *et al.*^2012^), the extremely sparse distribution of RCs across distantly related bacterial lineages (Sousa *et al.*^2013^; Fischer, Hemp and Johnson 2016), and the longstanding observation that chlorophototrophy occurs in combination with different CO_2_ fixation pathways—and sometimes in obligately aerobic heterotrophs (Table 1). Rather than having been imported into cyanobacteria, the export hypothesis has it that functional RCs were distributed among lineages as a result of modular export from the architects of chlorophototrophy—the cyanobacterial lineage.

### Possible reductive RC evolution

Finally, were the origin of RCs in all current anoxygenic photosynthetic lineages due to horizontal gene transfer, it would also directly explain why loss of chlorophototrophy is rarely observed among the free-living bacterial lineages in which it has taken hold. Absence of chlorophototrophy among most but not all bacterial (and archaeal) lineages is not the result of rampant loss, it is the result of not having acquired the trait. As a case in point, if there are free-living secondarily non-photosynthetic cyanobacterial lineages in nature, they are likely to be very rare, if they exist at all, based on metagenomic analyses published so far. This evidently is because once the GOE event occurred—the greatest ecological niche expansion in Earth's history—and organisms evolved into committed chlorophototrophic or chemoheterotrophic lifestyles, loss of chlorophototrophy would mean that any newly created, non-phototrophic mutant would have to displace a more highly evolved heterotroph from an existing ecological niche, an improbable event. For example, the conditions that would provide a selective advantage for a cyanobacterium that had lost the capacity for photoautotrophic growth would be highly unusual and very rare; indeed, only two closely related examples have been identified to date. Zehr and coworkers have described two variants of ‘*Candidatus* Atelocyanobacterium thalassa’, which are photoheterotrophic cyanobacterial symbiont partners of prymnesiophyte algae that have lost PSII and RuBisCO, but which have retained nitrogenase and PSI (Thompson *et al.*^2012^; Bombar *et al.*^2014^). These are examples of reductive evolution of photosynthesis within the cyanobacterial lineage.

How far can reductive evolution of photosynthesis go? The traditional interpretation of the homodimeric RCs of GSB and heliobacteria is that they reflect an ancient state of RCs. If they are the result of export from cyanobacteria, they might well be relicts of a very ancient transfer (Gisriel *et al.*^2017^), and in that sense molecular fossils from the early evolution of chlorophototrophy. This seems more likely than the alternative scenario that the heliobacterial RC arose by reductive evolution from a cyanobacterial PSI. We note in passing, however, that the concept of reductive evolution, a recurrent theme in molecular and genomic evolution (Ku *et al.*^2015^; Albalat and Cañestro 2016), is not widely discussed in the context of the evolution of chlorophototrophy. Nevertheless, several examples exist, primarily among the metabolically flexible *Proteobacteria* (for example, Zheng *et al.*^2012^; Keppen *et al.*^2013^; Koblížek *et al.*^2013^; Kopetjka *et al.*^2017^).

## CONCLUSIONS

From comparative physiology, we arrive at a list of main logical inferences about photosynthesis evolution as follows, all to be understood as conditional sentences, but written as declarative statements for readability. Life started off chemolithoautotrophically, with primary production at geological sources of H_2_ (hydrothermal vents). Flavin-based electron bifurcation was used by the first autotrophs for the reduction of low-potential Fd to produce Fd_red_, the essential electron donor in anaerobic pathways of CO_2_ fixation. Chl biosynthesis was the decisive invention for the origin of chlorophototrophy, which likely occurred in an anaerobe that had cobalamin, heme, cytochromes and quinones, and probably was capable of chemoheterotrophic growth. Chlorophototrophy evolved under low intensity hydrothermal-light conditions, in the midst of chemolithoautotrophic primary production, and where the photobiologically damaging effects of high-light flux and ultraviolet radiation were absent.

The first photochemically active pigments were possibly Zn-tetrapyrroles such as Zn-PPIX, the excited triplet of which (^3^Zn-PPIX) is strongly reducing (–1600 mV) with a long half-life (7–15 ms). The function (and physiological advantage) of the first chlorophototrophic electron transfer chain was Fd reduction via FeS clusters in RC1 with electrons from H_2_S, as occurs in modern-day GSB (for example, *Chlorobium* sp.). This freed primary production from H_2_ exhalation at vents. Photothioautotrophy, first with one RC then with two, was the physiological and evolutionary bridge between H_2_-dependent chemolithoautotrophy involving flavin-based electron biufurcation and chlorophototrophic oxidation of metal ions, both soluble and in the OEC. Photothiotrophy was a globally widespread (and the predominant) source of primary production in the photic zone of Archean oceans and was a trait of protocyanobacteria, with sulfate (sulfite) reducers regenerating H_2_S from photooxidized products (sulfur, sulfite and thiosulfate), giving rise to fluctuating mass-dependent sulfur isotope fractionation prior to the GOE. Whiffs of oxygen prior to the GOE reflect the existence of a strong oxidant in the oceans, which we suggest stems from an RC2 ancestor prior to the evolution of water oxidation.

Modern cyanobacterial photothiotrophy involves two different kinds of H_2_S oxidation that would permit, via gene regulation, and physiological modulation of the quinone pool redox state in the photosynthetic membrane: a two-electron reaction (to form S^0^) involving SQR and RCI, and a six-electron reaction (to form SO_3_^2–^/S_2_O_3_^2–^). Cyanobacterial ChlF is a relict from gene duplication that possibly gave rise to an ancestral homodimeric RC2. Photosynthesis arose in a cyanobacterial progenitor, and Chl *a* is the ancestral Chl. All anoxygenic chlorophotosynthetic lineages characterized so far are the result of acquisition via horizontal gene transfer of one RC and Chl biosynthesis, accompanied or not by a CO_2_ fixation pathway, via gene export from the cyanobacterial lineage, in some cases through intermediate lineages. Physiology speaks to the direction of those transfers but not to their geological timing. Prior to the origin of the OEC, reductants for CO_2_ fixation (H_2_ in cells without Chl and H_2_S in chlorophototrophs) were probably limiting for marine primary production.
